# Lateral metabolome study reveals the molecular mechanism of cytoplasmic male sterility (CMS) in Chinese cabbage

**DOI:** 10.1186/s12870-023-04142-w

**Published:** 2023-03-07

**Authors:** Huiju Yang, Mingwei Chen, Jingfeng Hu, Mei Lan, Jiangming He

**Affiliations:** 1 Lijiang Teachers College, Lijiang, 674199 China; 2 Institute of Horticultural Crops, Yunnan Academy of Agricultural Sciences, Kunming, 650205 China

**Keywords:** Chinese cabbage, Cytoplasmic male sterility, Metabolome

## Abstract

**Background:**

Chinese cabbage is one of the most widely grown leafy vegetables in China. Cytoplasmic male sterility (CMS) is a maternally inherited trait that produces abnormal pollen during anther development, which is commonly seen in cruciferous vegetables. However, the molecular mechanism of Chinese cabbage CMS is not clear. In this study, the metabolome and hormone profiles of Chinese cabbage male sterile line (CCR20000) and sterile maintainer line (CCR20001) were analyzed in flower buds during normal stamen development and abnormal stamen development, respectively.

**Results:**

A total of 556 metabolites were detected based on UPLC-MS/MS detection platform and database search, and the changes of hormones such as auxin, cytokinins, abscisic acid, jasmonates, salicylic acid, gibberellin acid and ethylene were analyzed. The results showed that compared with the male fertile line (MF), the male sterile line (MS) significantly decreased the content of flavonoids and phenolamides metabolites in the stamen dysplasia stage, accompanied by a large accumulation of glucosinolate metabolites. Meanwhile, the contents of GA9, GA20, IBA, tZ and other hormones in MS were significantly lower than those in MF strains. Further, by comparing the metabolome changes of MF and MS during stamen dysplasia, it was found that flavonoid metabolites and amino acid metabolites were distinctly different.

**Conclusions:**

These results suggest that flavonoids, phenolamides and glucosinolate metabolites may be closely related to the sterility of MS strains. This study provides an effective basis for further research on the molecular mechanism of CMS in Chinese cabbage.

**Supplementary Information:**

The online version contains supplementary material available at 10.1186/s12870-023-04142-w.

## Background

Chinese cabbage belongs to the large cruciferous family and is one of the most widely grown leafy vegetables in China [1]. The crop is so abundant in germplasm that it is currently available year-round [2, 3]. Since Chinese cabbage is a cross-pollinated crop, its heterosis is strong [4]. Self-incompatibility and male sterility are important means to exploit heterosis in cruciferous vegetables, and CMS is one of the most widely used systems [5–7]. CMS is a maternally inherited trait that has been found in more than 150 plants [8]. It is associated with abnormal reorganization of the mitochondrial genome, which is essential for the production of ATP through the mitochondrial electron transport chain of the respiratory complex [9–11]. Mitochondria are the main organelles that generate reactive oxygen species (ROS), and mitochondrial genome reorganization often leads to energy deficit and abnormal accumulation of ROS in plant anthers [12, 13]. Association analysis of a large number of mitochondrial DNA fragments with CMS revealed that abnormal chimeric genes were formed by frequent intra- or intermolecular genomic recombination, resulting in new gene expression patterns and even new genes [14, 15]. Due to the identical structure, most chimeric genes contain a subunit of the ATP synthase gene or cytochrome oxidase gene and open reading frame (ORF), which may disrupt mitochondrial function at critical moments in anther development, leading to CMS [15].

Numerous reports indicate that abnormal ROS levels and lack of ROS scavenging systems are associated with male sterility in crops [16–19]. There are two ROS scavenging systems in plants [20, 21]. The enzymatic ROS scavenging system usually consists of enzymes such as superoxide dismutase (SOD), catalase (CAT), peroxidase (POD), all of which are aberrantly expressed during pollen development in plants with CMS [22, 23]. The inactive ROS scavenging system is mainly composed of secondary metabolites, such as ascorbic acid (AsA) and glutathione (GSH), anthocyanins, flavonoids and phenolamides, which are major cellular redox buffers. Phenolamides are widely distributed in plants, usually as the main phenolic components in reproductive organs and seeds, and have specific functions in plant development and defense [23]. In addition, these metabolites play critical roles in pollen development and protection from ROS stress [24–27].

In addition, flavonoids such as flavonols (e.g. kaempferol and quercetin) are required for pollen vitality, germination, and tube development in maize, petunia, tomato, and tobacco [28, 29]. Mutations in flavonoid biosynthesis pathway genes such as FLS, F3H, CHS, and CHI may have extensive effects on pollen development, seed production, and male sterility in several plant species [28–30]. Application of flavonols exogenously restores seed production in maize CHS mutants and pollen-tube development in petunia CHS mutants [31, 32]. Moreover, Male Sterility (MS)-related metabolome and transcriptome research conducted in recent years indicate that flavonoid production plays a significant role in MS [33–36]. This indicates that flavonoids may play a crucial role in CMS.

Callose is a crucial component of sexual reproduction in plants because it creates the mother cell wall and the zygote wall of big spores. Callose inhibits agglomeration and fusion between cells when microspores are discharged, as well as premature growth and rupture of growing microspores. In the anthers of Chinese cabbage, callose is unable to be degraded in time during the late stage of tetrad growth of microspore mother cells, leading to defective tetrad formation and increased tapetum cell vacuolation. Anthers abort entirely. [37, 38]. Transcriptome studies have shown that a large number of genes related to pollen development, pollen tube growth, pollen wall development, plant hormones and transcription factors are involved in the physiological process of male sterility in Chinese cabbage [37, 38]. Alterations in phytohormone signaling, lipid metabolic transport, carbohydrate metabolism, and amino acid metabolism occur throughout the growth of Chinese cabbage anthers; hence, substantial changes in metabolites may be one of the key characteristics of CMS(CCR20000) [39–41].

However, the current research on the molecular mechanism of Chinese cabbage CMS is still incomplete. Metabolomics is a powerful tool to understand the mechanisms of metabolic regulation during flower, anther, and pollen development. In this study, we compared the buds in Chinese cabbage between a male sterile line (CCR20000) and a sterile maintainer line (CCR20001). The overall metabolome and hormone group changes in normal stamen development and abnormal stamen development.

## Results

### Metabolome analysis of Chinese cabbage buds

**Fig. 1:**
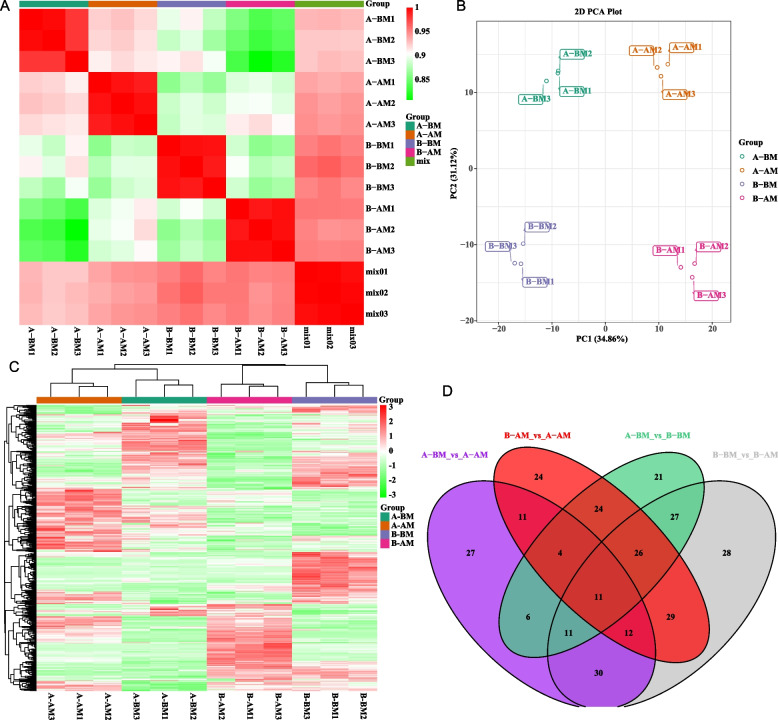
Quality control and analysis of metabolome data of Chinese cabbage CMS line and maintainer line in flower buds in normal stamen development and abnormal stamen development. **A**: Pearson correlation analysis between metabolome samples. **B**: PCA analysis between metabolome samples. **C**: Global content heatmap of differential metabolites between different samples of the metabolome. **D**: Venn diagram for the comparison of differential metabolites between different subgroups of the metabolome. (“A” and “B” represent cytoplasmic male sterile line CCR20000 and its male sterile maintainer line CCR20001, respectively. “AM” and “BM” represent flower buds at the stage of stamen dysplasia (SSD) and normal stamen development stage (NSD))

In this project, 12 samples were selected and divided into four groups, each with three biological replicates for metabolic research (Fig. 1A). Closer inspection of the data showed that R^2^ > 0.95, indicating that each line has good repeatability. PCA analysis of metabolite content showed that the samples in each group maintained good separation, and the samples within the group had good reproducibility (Fig. 1B). As can be seen from the global content heatmap, all metabolites had significant phasic and strain-specific expression characteristics, and the magnitude of metabolite content changes was stable between groups (Fig. 1C). Metabolite analysis identified 556 metabolites in CMS line CCR20000 and its maintainer line CCR20001, which include amino acid derivatives, flavonoid metabolites, alkaloids, amino acids, anthocyanins, coumarins, etc. Further differential analysis showed that there were 11 metabolites with differential co-expression among the four groups, and the number of repeats of differential metabolites between different groups was small (Fig. 1D). This may indicate that the regulation and changes of metabolic pathways in the stamen development process of the CMS line and its maintainer line are quite different.

### Metabolite changes during abnormal stamen development in Chinese cabbage CMS line and its maintainer line

**Fig. 2:**
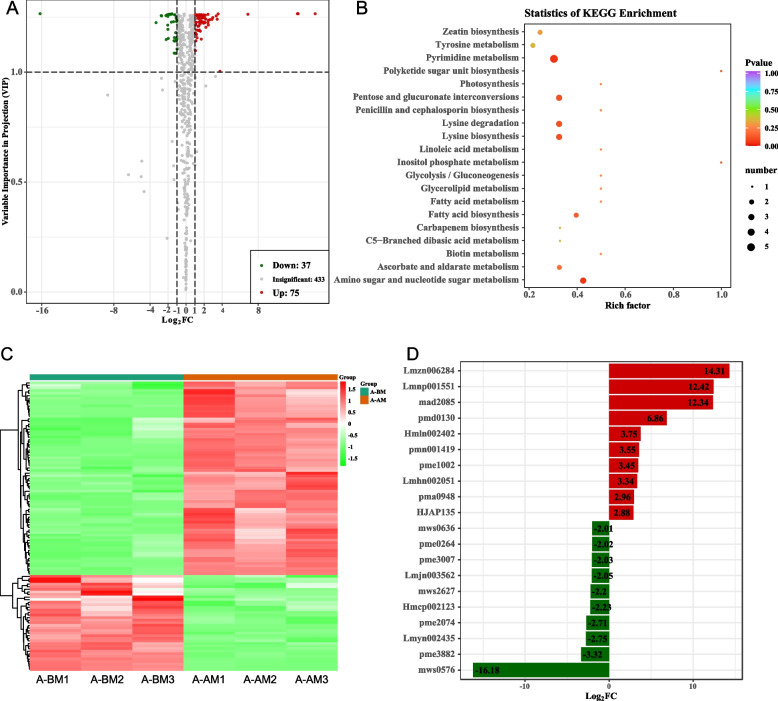
Metabolite changes during abnormal stamen development in Chinese cabbage CMS line and its maintainer line. **A**: Volcano plot for differential metabolite comparison. **B**: KEGG enrichment analysis of differential metabolites. **C**: Content heat map of differential metabolites. **D**: 20 differential metabolites with the most significant changes

In order to further analyze the differences in metabolomes of the CMS line and its maintainer line during stamen development, we conducted a differential analysis of the metabolome of the flower buds at the stamen dysplasia stage (SSD) between the CMS line and the maintainer line. Differential analysis identified a total of 69 up-regulated differential metabolites and 72 down-regulated differential metabolites between CMS lines and maintainer line (Fig. 2A). Functional analysis indicated that these differential metabolites were closely related to metabolic pathways such as arginine and proline metabolism, flavonoid biosynthesis, plant hormone signal transduction, flavone and flavonol biosynthesis (Fig. 2B). As shown in Fig. 2C, both these up-regulated differential metabolites and down-regulated differential metabolites exhibited specific expression changes. The changes in pmn001536 (Tetra-O-galloyl Methyl gallate) were most pronounced among the up-regulated differential metabolites, while changes in pmb0489 (N-hexosyl-p-coumaroyl putrescine) were most significant in down-regulated metabolites (Fig. 2D).

**Fig. 3:**
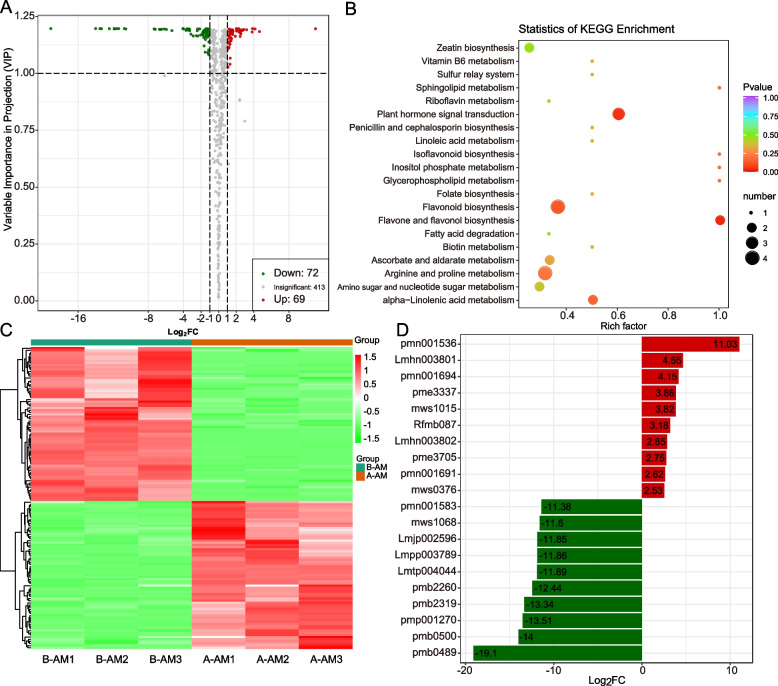
Dynamic changes of metabolome during abnormal stamen development in Chinese cabbage CMS line. **A**: Volcano plot for differential metabolite comparison. **B**: KEGG enrichment analysis of differential metabolites. **C**: Content heat map of differential metabolites. **D**: 20 differential metabolites with the most significant changes

In addition, we conducted a differential analysis of metabolites throughout the NSD and SSD stages of the CMS line and the maintainer line, respectively. Results indicated 112 differential metabolites between NSD and SSD in the CMS line (75 were up-regulated in SSD and 37 were down-regulated) (Fig. 3A, Table S1), whereas analysis of alterations in metabolites revealed 174 differential metabolites between NSD and SSD in the maintainer line (70 up-regulated in SSD, 104 down-regulated) (Table S2). Functional analysis showed that these differential metabolites between NSD and SSD in the CMS line were closely related to pyrimidine metabolism, pentose and glucuronate interconversions, amino sugar and nucleotide sugar metabolism, lysine biosynthesis and other metabolic pathways (Fig. 3B). As shown in Fig. 3C, both of these up-regulated differential metabolites and down-regulated differential metabolites exhibited specific expression changes. The change of Lmzn006284 (2α-hydroxyursolic acid) was the most obvious among the up-regulated differential metabolites, while the most significant change of mws0576 (3-Hydroxybutyrate) was among the down-regulated metabolites (Fig. 3D). This suggests that changes in these metabolites may be more relevant to stamen development in CMS lines. After removing the influence of the developmental stage, 45 metabolites, including nine flavonoid and nine phenolic acid metabolites, were identified as specifically elevated in the CMS line (Table S3). These include Butin-O-glucoside, Naringenin-O-glucoside, Quercetin-3-O-(6"-O-acetyl)-galactoside and other anthocyanin glycosides, and their increased specificity in the CMS line may be one of the causes of male sterility. Furthermore, 16 metabolites were identified as being specifically down-regulated in CMS cell lines (Table S4). These included Indole-5-carboxylic acid*, indole-3-carboxylic acid, and other plumerane compounds, as well as 2-hydroxy-4-pentenyl glucosinolate, 3-methylpentyl glucosinolate, 6-methylsulfinylhexyl glucosinolate and other glucosinolates compounds. These findings suggest that the up-regulation of flavonoids and phenolic acid metabolites in CMS lines and the down-regulation of glucosinolates and plumerane metabolites may significantly contribute to the formation of the CMS of Chinese cabbage.

### Changes in hormone content in Chinese cabbage buds

**Fig. 4:**
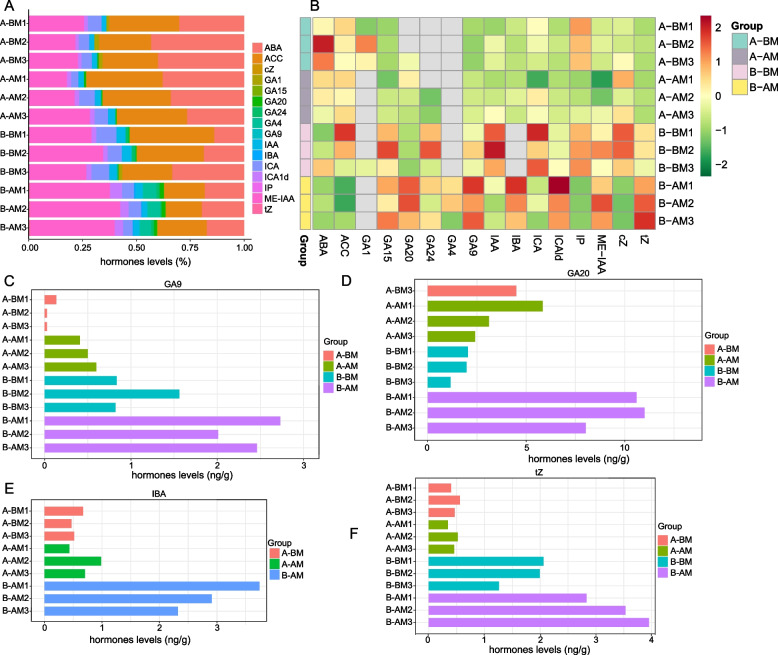
Determination and changes of hormone content in Chinese cabbage CMS line and its maintainer line during abnormal stamen development. **A**: Distribution of hormones in all samples. **B**: Heat map of changes in hormone content in all samples. **C**-**F** The content changes of GA9, GA20, IBA and tZ in Chinese cabbage CMS line and its maintainer line during abnormal stamen development, respectively

Plant hormones refer to trace organic substances synthesized in plants, usually transported from the synthesis site to the action site, regulating plant growth and development. The regulation of plant hormones on plant physiological activities involves changes in their own concentrations. Mastering the changes in the composition and content of hormones in plants will help to discover more interactions between plant hormones, and then explore the possible mechanism of action. In order to explore the changes of hormone content in flower buds of CMS line and maintainer line during stamen development, we analyzed the hormone content of 12 samples corresponding to metabolome samples. In this study, a total of 16 plant hormones, including auxin, cytokinin (CKs), abscisic acid (ABA), jasmonic acid (JAs), salicylic acid (SA), gibberellins (GAs), ethylene (ETH) and other categories, were detected by the LC–MS/MS platform-based plant hormone analysis method. Among them, Methyl indole-3-acetate (ME-IAA), ABA, 1-Aminocyclopropane carboxylic acid (ACC, as precursor of ethylene) were the main hormone types in Chinese cabbage buds (Fig. 4A). However, different types of hormones also showed dynamic changes in their contents at different stages of stamen development in the CMS and maintainer lines (Fig. 4B). Among them, Gibberellin A9(GA9), Gibberellin A20(GA9), trans-Zeatin(tZ) and 3-Indolebutyric acid (IBA) showed the most obvious changes. The contents of GA9, GA20, tZ and IBA were significantly lower in the CMS line than in the maintainer line during abnormal stamens development (Fig. 4C ~ F).

### Differences in metabolite changes of stamen dysplasia in Chinese cabbage MS and MF

**Fig. 5:**
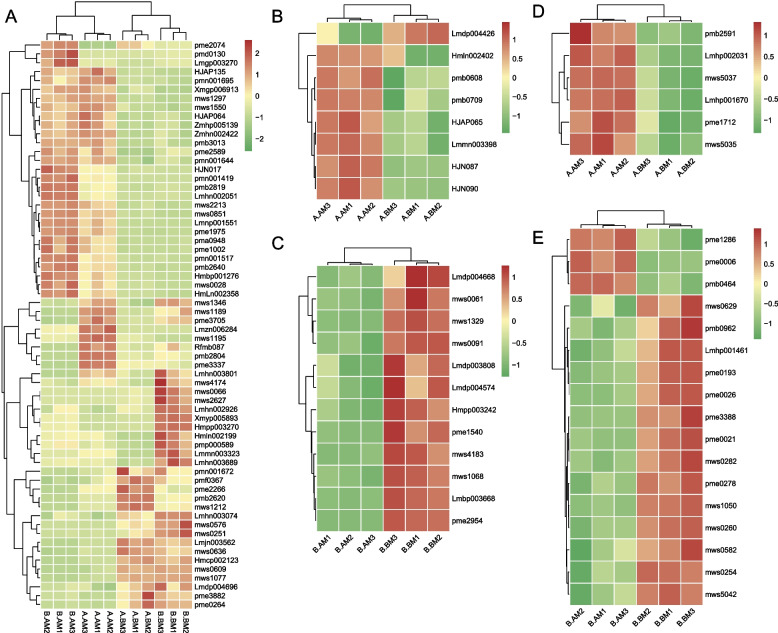
Changes of metabolites during abnormal stamen development in Chinese cabbage MS and MF. **A** Differential metabolites common to the CMS line (compared to the normal stamen development stage of the CMS line) and its maintainer line (compared to the maintainer line to the normal stamen development stage) during the period of abnormal stamen development. **B**-**C** Changes in the content of flavonoids in the CMS line and the maintainer line, respectively. **D**-**E** the content changes of amino acid compounds in the CMS line and the maintainer line, respectively

In order to further investigate the commonalities and differences in the metabolome changes of the CMS line and its maintainer line during stamen development, we compared the CMS line and its maintainer line. A total of 64 common differential metabolites were found, but the variation rules of these common differential metabolites in the CMS line and the maintainer line were not exactly the same (Fig. 5A). These common differential metabolites mainly include amino acid derivatives and flavonoid metabolites. In addition, metabolites with specific differences between the CMS line and the maintainer line were also counted. Among them, the metabolites of flavonoids and amino acids had the most significant differences. Seven flavonoid metabolites (kaempferol-3-o-(6''-malonyl)-galactoside*, chrysoeriol-o-malonylhexoside, quercetin-7-o-malonylhexosyl-hexoside, kaempferol-malonyl-3-o-glucoside*, kaempferol acetyl-glucoside, naringenin-o-glucoside* and butin-o-glucoside*) were up-regulated during the period of stamen dysplasia in the CMS line, while 12 flavonoid metabolites (such as kaempferol-o-feruloylglucosid-o-glucoside, quercetin-3-o-β-d-galactoside (hyperin)*, quercetin-7-o-glucoside* and so on) decreased in the period of stamen dysplasia in the maintainer line (Fig. 5B-C). Similarly, we found that 6 amino acid metabolites (acetyltryptophan, leu- leu, alanylleucine, val-leu, l-saccharopine and leucylphenylalanine) were up-regulated in CMS lines during stamen dysplasia, while 14 amino acid metabolites (including asp-phe, lysine butyrate, pro-leu, and so on) decreased in maintainer lines during stamen dysplasia (Fig. 5D-E). Our analysis results showed that the changes of flavonoids and amino acids metabolites in the CMS line and maintainer line during the period of stamen dysplasia were diametrically opposite, which may indicate that the changes of these metabolites are related to the molecular mechanism of the sterility of the CMS line.

### Differences of flavonoids and phenolamine metabolites in Chinese cabbage during MS and MF stamen dysplasia

**Fig. 6:**
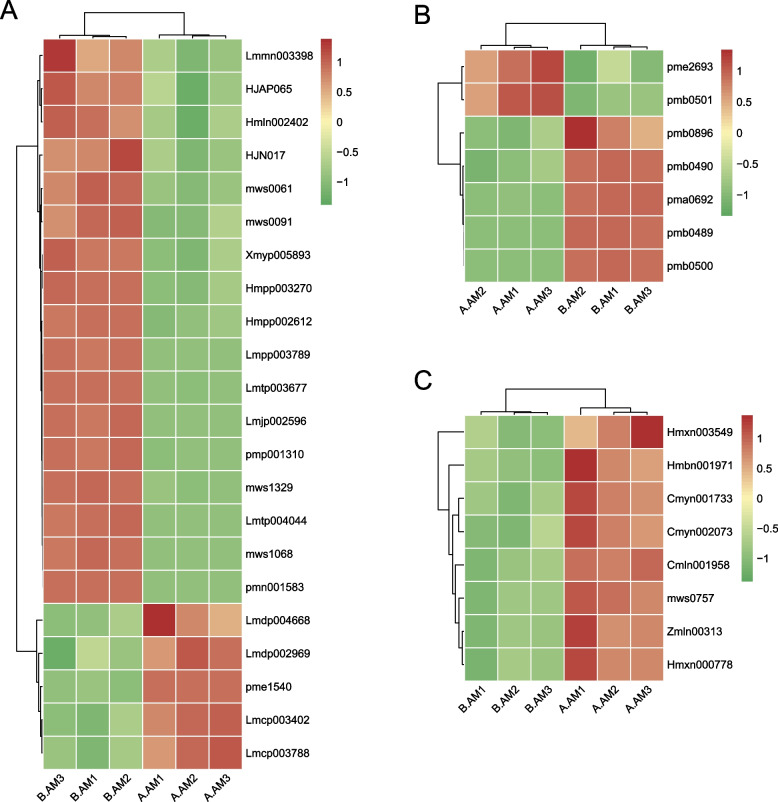
Changes of flavonoids, phenolamides and glucosinolates metabolites during MS and MF stamen dysplasia in Chinese cabbage. **A**: Changes in flavonoid metabolites. **B**: Changes in phenolamine metabolites. **C**: Changes in glucosinolate metabolites

To further analyze the differences between the CMS line and its maintainer in stamen dysplasia, the differential metabolites were screened. The results showed that phenolamides, flavonoids and glucosinolate metabolites were among the most different. A total of 22 flavonoids, 7 phenolamides, and 8 glucosinolate differential metabolites were identified between the CMS line and its maintainer. Among them, 17 flavonoid differential metabolites were down-regulated and five flavonoids (kaempferol-o-feruloylglucosid-o-glucoside, myricetin 3-o-galactoside, isorhamnetin-3-o-neohesperidoside, tamarixin and isotamarixin) were up-regulated in CMS lines (Fig. 6A). Likewise, five phenolamides differential metabolites (n-feruloylputrescine*, p-coumaroylputrescine, n(1),n(8)-bis(p-coumaroyl)spermidine, n-hexosyl-p-coumaroyl putrescine and n-p-coumaroyl-n'-feruloylputrescine) were up-regulated and two phenolamides (n-acetylputrescine and agmatine) were down-regulated in the CMS line (Fig. 6B). In addition, all glucosinolate differential metabolites (including 1,4-Dimethoxyglucobrassicin, m-Methoxybenzyl glucosinolate, 1-Methylpropyl glucosinolate,, etc.) were up-regulated in the CMS line (Fig. 6C). Changes in these metabolites may be related to the antioxidant system of flower buds in Chinese cabbage, and changes in the antioxidant system may cause changes in ROS levels.

## Discussion

Although recent studies have reported metabolite changes during flower, anther, and pollen development in many CMS crops, the changes and molecular mechanisms of the metabolome in CMS plants are not yet fully understood [42, 43]. In this study, we compared the overall changes of metabolome and hormone profiles in flower buds between the male sterile line (CCR20000) and the sterile maintainer line (CCR20001) of Chinese cabbage at both normal and abnormal stamen development stages. Compared with the maintainer line, more metabolites in the CMS line were down-regulated, which may lead to pollen abortion in the Chinese cabbage CMS line. At the same time, a large number of amino acids and amino acid derivatives, organic acids and other metabolites were significantly down-regulated in CMS lines. Downregulation of these metabolites may impede the normal metabolism and energy supply of cells, further affecting pollen development [35]. In addition, flavonoids and phenolamides are the two most differentiated metabolites, and many studies suggest that they may contribute to ROS scavenging and pollen development [24, 26, 27].

Abnormal ROS status is associated with pollen abortion in many CMS plants [31, 44]. A total of 29 differential metabolites were found to be related to the oxidative resistance of Chinese cabbage, including 22 flavonoids and 7 phenolamides. Flavonoids are one of the powerful secondary metabolites that protect plants from ROS stress [20]. In plants, flavonoids can be oxidized by POD to scavenge H_2_ O_2_ in the phenol/AsA/POD system [45]. In this study, 17 of the 22 differential flavonoids in flower buds of Chinese cabbage CMS line and their maintainers were down-regulated (including Cyanidin-3-O-(6'-p-Coumaroylglucoside), Quercetin-3-O- β-D-Galactoside (Hyperin)*, Quercetin-3-O-β-D-glucoside (Isoquercitrin)*, etc.). Quercetin is one of the most important forms of flavonoids in plants, and previous studies in petunia and tobacco have shown that it is essential for the germination and growth of pollen tubes [32, 46]. Flavonoids are present in the pollen of many plants and play an important role in pollen development, especially in germination and tube growth [47, 48].

The production of flavonoids relies on chalcone synthase (CHS), the first enzyme in the flavonoid pathway [32, 46, 47]. Studies have shown that antisense inhibition of CHS causes pollen sterility in many plants, including petunia, maize, and tomato. In a previous study, downregulation of CHS involved in the flavonoid synthesis pathway was detected during flower bud development in Chinese cabbage CMS [48]. In the present study, we observed a substantial decrease in flavonoid content during flower bud development in the CMS line. Downregulation of CHS in CMS lines may reduce flavonoid synthesis, which thereby diminishes ROS scavenging efficiency, leading to ROS burst and pollen abortion [24, 26, 27]. Decreased flavonoid content may also lead to the failure of pollen germination in sterile Chinese cabbage lines [26, 27].

Phenolamides are products of polyamine catabolism and are a major group of secondary metabolites abundant in reproductive organs of flowering plants [49, 50]. We detected seven phenolamides in the Chinese cabbage bud CMS line, including N-acetylputrescine, p-Coumaroylputrescine, N-p-Coumaroyl-N'-feruloylputrescine, etc. In this study, a total of five phenolamides were down-regulated in Chinese cabbage CMS lines. Phenolamides are considered essential components for flower induction and development and have been detected in the anthers and pollens of dicots [51–53]. Phenolamides accumulate first in upper leaves and later in floral organs, which are essential for pollen development, vigor or germination [23, 54, 55]. In maize, their absence in the anthers results in male sterility. Phenolamides are also known to have antioxidant and free radical scavenging properties, and they are substrates for PODs that eliminate H_2_O_2_ [1, 56]. Blocked synthesis of phenolic amides may limit ROS scavenging and affect pollen development, pollen viability and germination [23, 54, 55]. This suggests that the overall down-regulation of phenolamides in CMS lines of Chinese cabbage may lead to the weakened antioxidant activity and abnormal pollen development of CMS lines.

Gibberellins and polyamines are advantageous to the development of male organs, and the gibberellin concentration in the juvenile panicles and anthers of CMS rice sterile plants is much lower than that of the comparable fertile plants [57, 58]. This investigation revealed that the content of GA9 and GA20 in the CMS line at the aberrant stamen stage was much lower than in the maintainer line, suggesting that the function of GA in the CMS of Chinese cabbage may be comparable to that of other species [59]. Ethylene and abscisic acid are usually seen as having an inhibiting impact on the male growth of plants, and environmental conditions such as temperature may influence CMS through ABA hormone level variations [60]. The changing pattern of ethylene precursor ACC and ABA content in the CMS series is opposite to that of the GA series, which suggests that ethylene and ABA play a similar function in Chinese cabbage CMS. Similarly, the cytokinin content of most CMS plants was typically greater than that of maintainer lines, however in this research, the cytokinin (IP, cZ, tZ) levels were lower in the CMS lines than in the maintainer lines. We hypothesized that the lower cytokinin content in the CMS line may interfere with the normal development of anthers and pollen, therefore generating MS. Moreover, inadequate polysaccharide and starch content in flower buds would inhibit energy metabolism, resulting in insufficient cell production capacity, impeding the development of different floral components, and causing miscarriage. Flower growth is dependent on the presence of the amino acid proline in the cells of the developing flower. It may supply essential carbon and nitrogen sources for flower growth and can be directly utilized for protein synthesis. The decrease of different carbohydrate metabolites and free amino acids in the CMS line may interfere with the proper development of spores in Chinese cabbage, causing CMS.

## Conclusions

The metabolome and hormone profiles of Chinese cabbage male sterile line (CCR20000) and sterile maintainer line (CCR20001) were analyzed in flower buds during normal stamen development and abnormal stamen development, respectively. Our findings demonstrate that the MS significantly decreased the content of flavonoids and phenolamides metabolites in the stamen dysplasia stage, as compared to the MF, and was also accompanied by a large accumulation of glucosinolate metabolites. In the meantime, MS strains had significantly lower levels of GA9, GA20, IBA, tZ, and other hormones than MF strains did. Additionally, it was discovered that the changes in flavonoid and amino acid metabolites between MF and MS during stamen dysplasia were significantly different. These findings imply that the sterility of MS strains may be closely related to flavonoids, phenolamides, and glucosinolate metabolites. The findings of this study serve as a strong foundation for future investigations into the molecular mechanism of CMS in Chinese cabbage.

## Methods

### Plant material

In March 2020, cytoplasmic male sterile line CCR20000 (A) and its male sterile maintainer line CCR20001 (B) of Chinese cabbage lines were collected from the experimental base in Songming, Kunming city, Yunnan province, China. Chinese cabbage was planted in the field with normal water and fertilizer management. These materials identified by Jiangming He (Yunnan Academy of Agricultural Sciences). The two different materials were collected at two different developmental stages based on stamen development. The size of flower buds was less than 1.5 mm in the period of normal stamen development and more than 3 mm in the period of abnormal stamen development. The maintainer line (male fertile) was gathered at the same period as the sterile line. Each group was repeated three times, and metabolome and hormone analysis were performed respectively. The materials involved in the experiment were cultivated, collected and licensed by Yunnan Academy of Agricultural Sciences. Our experimental research complied with local legislation, national and international guidelines.

### Metabolite extraction

The freeze-dried leaf was crushed using a mixer mill (MM 400, Retsch) with a zirconia bead for 1.5 min at 30 Hz. A hundred milligrams of powder was weighed and extracted overnight at 4℃ with 0.6 ml 70% aqueous methanol. Following centrifugation at 10, 000 g for 10 min, the extracts were absorbed (CNWBOND Carbon-GCB SPE Cartridge, 250 mg, 3 ml; ANPEL, Shanghai,China, www.anpel.com.cn/cnw) and filtrated (SCAA-104, 0.22 μm pore size; ANPEL, Shanghai, China, http://www.anpel.com.cn/) before UPLC-MS/MS analysis.

### UPLC-MS/MS Analysis

The UPLC-MS/MS analysis according to Michopoulos et al. 's method with minor modification [61]. The sample extracts were analyzed using an UPLC-ESI–MS/MS system (UPLC, Shim-pack UFLC SHIMADZU CBM30A system, www.shimadzu.com.cn/; MS, Applied Biosystems 4500 Q TRAP, www.appliedbiosystems.com.cn/). The separation method of UPLC was used on an Agilent SB-C18 (1.8 µm, 2.1 mm*100 mm). The mobile phase consisted of pure water with 0.1% formic acid (solvent A), and acetonitrile (solvent B). Sample measurements were performed with a gradient program that employed the starting conditions of 95% A and 5% B. Within 9 min, a linear gradient to 5% A and 95% B was programmed, and a composition of 5% A and 95% B was kept for 1 min. Subsequently, a composition of 95% A and 5.0% B was adjusted within 1.10 min and kept for 2.9 min. The column oven was set to 40 °C; while the injection volume was 4 μl. The effluent was alternatively connected to an ESI-triple quadrupole-linear ion trap (QTRAP)-MS.

### Metabolite characterization and quantification

Based on the self-built database MWDB (metware database), the material is qualitatively based on the secondary spectrum information, and the isotopic signal is removed from the analysis, including K^+^ ions, Na^+^ ions, NH4^+^ ions. Repeated signals are considered as fragment ions that are other larger molecular weight substances themselves. Metabolite quantification was performed using triple quadrupole mass spectrometry in the multiple reaction monitoring (MRM) mode. In the MRM mode, the quadrupole first screens the precursor ions of the target substance, and excludes the ions corresponding to other molecular weight substances to initially eliminate interference. The precursor ions are ionized by the collision cell and then break off to form many fragment ions, which pass through the triple quadruple. The stage rod filter selects a required characteristic fragment ion, eliminates the interference of non-target ions, and makes the quantification more accurate and repeatable. After obtaining the metabolite spectrum analysis data of different samples, the peak area integration is performed on the mass spectrum peaks of all substances, and the integration correction is performed on the mass spectral peaks of the same metabolite in different samples [62].

### Hormone content determination

Fresh plant materials were harvested, weighed, and immediately frozen in liquid nitrogen before being stored at -80 °C until required. Plant materials (50 mg fresh weight) were frozen in liquid nitrogen, ground into powder, and extracted with methanol/water/formic acid (15:4:1, V/V/V). Before LC–MS/MS analysis, the mixed extracts were evaporated to dryness using a nitrogen gas stream, reconstituted in 80% methanol (V/V), and filtrated (PTFE, 0.22 μm; Anpel). The sample extracts were analyzed using an LC–ESI–MS/MS system (HPLC, Shim-pack UFLC SHIMADZU CBM30A system, www.shimadzu.com.cn/; MS, Applied Biosystems 6500 Triple Quadrupole, www.appliedbiosystems.com.cn/). [63, 64] Using a Waters ACQUITY UPLC HSS T3 C18 (1.8 µm, 2.1 mm*100 mm) column, chromatographic separation was achieved at 40℃ with a flow rate of 0.35 mL/min. Solvent system consisted of water (0.05% acetic acid) and acetonitrile (0.05% acetic acid). The gradient program is as follows: 95:5 V/V at 0 min, 95:5 V/V at 1 min, 5:95 V/V at 8 min, 5:95 V/V at 9 min, 95:5 V/V at 9.1 min, and 95:5 V/V at 12 min. The injection volume was 2 μL. The effluent was alternatively connected to an ESI-triple quadrupole-linear ion trap (QTRAP)-MS.

### Metabolome bioinformatics analysis

The metabolites significantly regulated between groups were identified by VIP >  = 1 and absolute Log2FC (fold change) >  = 1. VIP values were extracted from the OPLS-DA results, which also contained score plots and permutation plots, using R package MetaboAnalystR [65]. The data underwent log transform (log2) and mean centering before OPLS-DA. To avoid overfitting, a permutation test (200 permutations) was carried out.

### KEGG enrichment analysis

The KEGG Compound database (http://www.kegg.jp/kegg/compound/) was used to annotate the identified metabolites. The annotated metabolites were then mapped to KEGG Pathway database (http://www.kegg.jp/kegg/pathway.html). Pathways with significantly regulated metabolites were mapped to be then fed into metabolite sets enrichment analysis (MSEA). Their significance was measured by hypergeometric test's *p*-values.

## Supplementary Information

**Additional file 1: Supplemental Table 1.** Differential metabolites between NSD and SSD in the CMS line.** Supplemental Table 2. **Differential metabolites between NSD and SSD in the maintainer line.** Supplemental Table 3.** Specifically elevated metabolites in the SSD stage of CMS lines.** Supplemental Table 4.** Specifically down-regulated metabolites in the SSD stage of CMS lines.

## Data Availability

The data that support the findings of this study are available from the author Jiangming He upon reasonable request.

## Abbreviations

ABA: Abscisic acid
ACC: 1-Aminocyclopropanecarboxylic acid
AsA: Ascorbic acid
ATP: Adenosine-triphosphate
CHS: Chalcone synthase
CHI: Chalcone isomerase
CAT: Catalase
CMS: Cytoplasmic male sterility
CKs: Cytokinin
CHS: Chalcone synthase
cZ: Cis-Zeatin
SSD: Stage of stamen dysplasia
ETH: Ethylene
FLS: Flavonol synthase
F3H: Flavanone-3-hydroxylase
GA: Gibberellin
GSH: Glutathione
IBA: Indole butyric acid
IP: Isoamyl alkenyl adenine
Jas: Jasmonic acid
KEGG: Kyoto encyclopedia of genes and genomes
ME-IAA: Methyl indole-3-acetate
MF: Male fertile line
MS: Male sterile line
MSEA: Metabolite sets enrichment analysis
MRM: Multiple reaction monitoring
NSD: Normal stamen development stage
ORF: Open reading frame
OPLS-DA: Orthogonal partial least squares-discriminant analysis
POD: Peroxidase
ROS: Reactive oxygen species
SA: Salicylic acid
SOD: Superoxide dismutase
tZ: Trans-zeatin
UPLC-MS/MS: Ultra performance liquid chromatography-tandem mass spectrometry
VIP: Variable importance in the projection
