# Supplementary material for: Lateral metabolome study reveals the molecular mechanism of cytoplasmic male sterility (CMS) in Chinese cabbage
Source: BMC Plant Biol. 2023 Mar 7;23:128. doi: 10.1186/s12870-023-04142-w (PMC9990347; doi:10.1186/s12870-023-04142-w)
Supplement: Supplementary file 1 — Additional file 1: Supplemental Table 1. Differential metabolites between NSD and SSD in the CMS line. Supplemental Table 2. Differential metabolites between NSD and SSD in the maintainer line. Supplemental Table 3. Specifically elevated metabolites in the SSD stage of CMS lines. Supplemental Table 4. Specifically down-regulated metabolites in the SSD stage of CMS lines. [file 12870_2023_4142_MOESM1_ESM.docx]

**Supplemental Table 1 Differential metabolites between NSD and SSD in the CMS line.**

| Index | Formula | Compounds | Class I | Class II | CAS | Level | A-BM1 | A-BM2 | A-BM3 | A-AM1 | A-AM2 | A-AM3 | VIP | Fold_Change | Log2FC | Type |
| --- | --- | --- | --- | --- | --- | --- | --- | --- | --- | --- | --- | --- | --- | --- | --- | --- |
| pme1975 | C3H4O4 | Malonic acid | Organic acids | Organic acids | 141-82-2 | A | 1.12E+06 | 9.56E+05 | 1.17E+06 | 3.24E+06 | 2.77E+06 | 2.57E+06 | 1.24E+00 | 2.64E+00 | 1.40E+00 | up |
| mws0576 | C4H8O3 | 3-Hydroxybutyrate* | Organic acids | Organic acids | 300-85-6 | A | 7.78E+05 | 6.49E+05 | 5.83E+05 | 9.00E+00 | 9.00E+00 | 9.00E+00 | 1.27E+00 | 1.34E-05 | -1.62E+01 | down |
| pme1730 | C4H6O4 | D-Erythronolactone | Organic acids | Organic acids | 15667-21-7 | A | 6.13E+05 | 6.78E+05 | 7.12E+05 | 3.40E+05 | 3.31E+05 | 2.83E+05 | 1.24E+00 | 4.76E-01 | -1.07E+00 | down |
| pma0134 | C4H8O4 | D(-)-Threose | Others | Saccharides and Alcohols | 95-43-2 | B | 4.11E+03 | 4.89E+03 | 9.32E+03 | 3.20E+04 | 3.01E+04 | 3.90E+04 | 1.21E+00 | 5.52E+00 | 2.46E+00 | up |
| mws0628 | C7H6O2 | 4-Hydroxybenzaldehyde | Phenolic acids | Phenolic acids | 123-08-0 | A | 3.86E+05 | 3.73E+05 | 3.69E+05 | 7.69E+05 | 7.40E+05 | 8.15E+05 | 1.26E+00 | 2.06E+00 | 1.04E+00 | up |
| mws0251 | C5H6N2O2 | Thymine | Nucleotides and derivatives | Nucleotides and derivatives | 65-71-4 | A | 1.60E+04 | 1.75E+04 | 9.28E+03 | 6.47E+03 | 5.12E+03 | 5.38E+03 | 1.14E+00 | 3.96E-01 | -1.33E+00 | down |
| pmb0501 | C5H14N4 | Agmatine | Alkaloids | Phenolamine | 306-60-5 | B | 4.40E+04 | 5.13E+04 | 5.49E+04 | 1.09E+05 | 8.54E+04 | 1.11E+05 | 1.21E+00 | 2.03E+00 | 1.02E+00 | up |
| pma0948 | C8H11NO | Hydroxyphenethylamine* | Others | Others | 7568-93-6 | B | 5.84E+04 | 7.05E+04 | 4.37E+04 | 4.00E+05 | 3.89E+05 | 5.57E+05 | 1.25E+00 | 7.80E+00 | 2.96E+00 | up |
| pme1002 | C8H11NO | L-Tyramine* | Amino acids and derivatives | Amino acids and derivatives | 51-67-2 | A | 1.76E+05 | 1.78E+05 | 8.81E+04 | 1.45E+06 | 1.42E+06 | 1.96E+06 | 1.24E+00 | 1.09E+01 | 3.45E+00 | up |
| mws2213 | C9H8O2 | Cinnamic acid | Phenolic acids | Phenolic acids | 140-10-3 | B | 1.87E+04 | 2.53E+04 | 1.40E+04 | 7.33E+04 | 6.69E+04 | 8.86E+04 | 1.22E+00 | 3.94E+00 | 1.98E+00 | up |
| mws0921 | C9H10O2 | p-Coumaryl alcohol | Phenolic acids | Phenolic acids | 3690-05-9 | A | 6.58E+04 | 6.00E+04 | 3.59E+04 | 3.31E+05 | 2.99E+05 | 3.82E+05 | 1.24E+00 | 6.26E+00 | 2.65E+00 | up |
| mws0851 | C8H15NaO2 | Sodium Valproate | Organic acids | Organic acids | 1069-66-5 | A | 6.64E+06 | 8.28E+06 | 9.70E+06 | 3.75E+07 | 3.46E+07 | 3.06E+07 | 1.25E+00 | 4.17E+00 | 2.06E+00 | up |
| pme2589 | C6H8O5 | Oxoadipic acid | Organic acids | Organic acids | 3184-35-8 | B | 1.12E+05 | 8.58E+04 | 9.11E+04 | 5.48E+05 | 4.10E+05 | 3.55E+05 | 1.24E+00 | 4.54E+00 | 2.18E+00 | up |
| mws0102 | C9H7NO2 | Indole-5-carboxylic acid* | Alkaloids | Plumerane | 1670-81-1 | B | 8.29E+05 | 7.61E+05 | 8.76E+05 | 4.43E+05 | 4.07E+05 | 3.35E+05 | 1.23E+00 | 4.80E-01 | -1.06E+00 | down |
| mws1417 | C9H7NO2 | Indole-3-carboxylic acid* | Alkaloids | Plumerane | 771-50-6 | B | 1.30E+06 | 1.23E+06 | 1.29E+06 | 6.96E+05 | 6.51E+05 | 5.47E+05 | 1.24E+00 | 4.96E-01 | -1.01E+00 | down |
| mws1550 | C6H11NO2S | S-Allyl-L-cysteine | Amino acids and derivatives | Amino acids and derivatives | 21593-77-1 | A | 5.15E+04 | 4.98E+04 | 7.49E+04 | 1.77E+05 | 1.54E+05 | 1.79E+05 | 1.23E+00 | 2.90E+00 | 1.54E+00 | up |
| mws1346 | C6H11NO4 | DL-2-Aminoadipic acid | Alkaloids | Alkaloids | 542-32-5 | B | 5.96E+05 | 5.41E+05 | 9.55E+05 | 2.12E+06 | 2.01E+06 | 1.71E+06 | 1.19E+00 | 2.79E+00 | 1.48E+00 | up |
| Lmnp001551 | C9H10O3 | 3-methoxy-4-hydroxy-acetophenone | Phenolic acids | Phenolic acids | - | B | 9.00E+00 | 9.00E+00 | 9.00E+00 | 6.06E+04 | 4.47E+04 | 4.27E+04 | 1.27E+00 | 5.48E+03 | 1.24E+01 | up |
| mws0467 | C9H10O3 | 3-(4-Hydroxyphenyl)-propionic acid | Phenolic acids | Phenolic acids | 501-97-3 | B | 1.27E+05 | 1.39E+05 | 1.10E+05 | 3.64E+04 | 3.44E+04 | 5.08E+04 | 1.23E+00 | 3.23E-01 | -1.63E+00 | down |
| Hmbp001276 | C8H8O4 | Gallacetophenone* | Others | Others | 528-21-2 | A | 3.61E+05 | 3.62E+05 | 3.55E+05 | 1.57E+06 | 1.23E+06 | 1.08E+06 | 1.25E+00 | 3.60E+00 | 1.85E+00 | up |
| mws0028 | C8H8O4 | Vanillic acid | Phenolic acids | Phenolic acids | 121-34-6 | B | 4.49E+04 | 5.19E+04 | 3.94E+04 | 2.61E+05 | 1.81E+05 | 1.48E+05 | 1.23E+00 | 4.33E+00 | 2.11E+00 | up |
| pme2598 | C8H8O4 | 3,4-Dihydroxybenzeneacetic acid* | Phenolic acids | Phenolic acids | 102-32-9 | A | 2.32E+05 | 2.59E+05 | 2.79E+05 | 7.50E+05 | 6.68E+05 | 7.54E+05 | 1.26E+00 | 2.82E+00 | 1.50E+00 | up |
| pme3009 | C6H6O6 | Trans-Citridic acid | Organic acids | Organic acids | 4023-65-8 | A | 5.07E+05 | 5.41E+05 | 6.19E+05 | 1.20E+06 | 1.09E+06 | 1.23E+06 | 1.25E+00 | 2.11E+00 | 1.08E+00 | up |
| pmf0367 | C9H6O4 | 5,7-Dihydroxychromone | Others | Others | 31721-94-5 | B | 2.95E+04 | 2.65E+04 | 2.35E+04 | 1.31E+04 | 1.62E+04 | 9.28E+03 | 1.15E+00 | 4.85E-01 | -1.04E+00 | down |
| mws1195 | C10H10O3 | Methyl p-coumarate* | Phenolic acids | Phenolic acids | 3943-97-3 | A | 3.07E+05 | 2.51E+05 | 3.00E+05 | 1.05E+06 | 9.72E+05 | 9.29E+05 | 1.26E+00 | 3.44E+00 | 1.78E+00 | up |
| mws1200 | C10H10O3 | Trans-4-Hydroxycinnamic Acid Methyl Ester* | Phenolic acids | Phenolic acids | 19367-38-5 | A | 8.95E+05 | 7.38E+05 | 8.14E+05 | 2.31E+06 | 2.12E+06 | 2.00E+06 | 1.25E+00 | 2.63E+00 | 1.39E+00 | up |
| pmb2795 | C10H10O3 | 4-Methoxycinnamic acid* | Phenolic acids | Phenolic acids | 830-09-1 | A | 4.92E+05 | 4.34E+05 | 4.88E+05 | 1.72E+06 | 1.54E+06 | 1.45E+06 | 1.26E+00 | 3.34E+00 | 1.74E+00 | up |
| Hmcp002123 | C10H8O4 | 6-Hydroxy-7-methoxycoumarin | Lignans and Coumarins | Coumarins | 776-86-3 | A | 7.32E+05 | 7.77E+05 | 6.35E+05 | 1.50E+05 | 1.38E+05 | 1.69E+05 | 1.26E+00 | 2.13E-01 | -2.23E+00 | down |
| pme3705 | C6H10O7 | D-Glucoronic acid* | Others | Saccharides and Alcohols | 6556-12-3 | B | 8.23E+04 | 7.45E+04 | 7.50E+04 | 2.82E+05 | 2.31E+05 | 2.23E+05 | 1.25E+00 | 3.17E+00 | 1.67E+00 | up |
| mws1189 | C6H10O7 | D-Galacturonic acid(Gal A)* | Organic acids | Organic acids | 685-73-4 | A | 7.97E+04 | 8.94E+04 | 9.19E+04 | 2.91E+05 | 2.81E+05 | 2.72E+05 | 1.26E+00 | 3.24E+00 | 1.69E+00 | up |
| Xmgn006542 | C10H10O4 | Dimethyl Phthalate | Others | Others | 131-11-3 | B | 8.62E+05 | 8.20E+05 | 8.08E+05 | 2.56E+05 | 2.34E+05 | 2.30E+05 | 1.26E+00 | 2.89E-01 | -1.79E+00 | down |
| pmb2640 | C12H24O2 | Lauric acid | Lipids | Free fatty acids | 143-07-7 | B | 1.01E+05 | 1.16E+05 | 1.28E+05 | 3.16E+05 | 3.02E+05 | 2.84E+05 | 1.25E+00 | 2.62E+00 | 1.39E+00 | up |
| mws5037 | C9H18N2O3 | Alanylleucine | Amino acids and derivatives | Amino acids and derivatives | 1999-42-4 | A | 8.35E+05 | 8.11E+05 | 1.09E+06 | 1.88E+06 | 1.81E+06 | 1.84E+06 | 1.22E+00 | 2.02E+00 | 1.02E+00 | up |
| Hmln002597 | C10H9NaO4 | Sodium ferulate | Others | Others | - | A | 6.80E+05 | 6.51E+05 | 6.56E+05 | 1.93E+05 | 1.77E+05 | 1.65E+05 | 1.26E+00 | 2.69E-01 | -1.89E+00 | down |
| mws1212 | C11H12O4 | Methyl ferulate | Phenolic acids | Phenolic acids | 2309-07-1 | A | 1.25E+07 | 1.18E+07 | 1.14E+07 | 4.05E+06 | 3.88E+06 | 3.91E+06 | 1.26E+00 | 3.32E-01 | -1.59E+00 | down |
| pmb2620 | C11H12O4 | 3,4-Dimethoxycinnamic acid | Phenolic acids | Phenolic acids | 14737-89-4 | A | 1.26E+07 | 1.29E+07 | 1.24E+07 | 4.28E+06 | 4.35E+06 | 4.10E+06 | 1.26E+00 | 3.36E-01 | -1.57E+00 | down |
| mws4174 | C8H15NO6 | N-Acetyl-β-D-mannosamine* | Others | Others | 7772-94-3 | B | 4.04E+04 | 4.02E+04 | 6.71E+04 | 9.03E+04 | 1.09E+05 | 1.16E+05 | 1.14E+00 | 2.13E+00 | 1.09E+00 | up |
| pme3882 | C9H12N2O5 | 2'-Deoxyuridine | Nucleotides and derivatives | Nucleotides and derivatives | 951-78-0 | B | 2.91E+04 | 5.65E+04 | 2.24E+04 | 4.08E+03 | 3.14E+03 | 3.57E+03 | 1.22E+00 | 9.99E-02 | -3.32E+00 | down |
| Lmhp001670 | C11H22N2O3 | Val-Leu | Amino acids and derivatives | Amino acids and derivatives | - | A | 3.80E+05 | 3.54E+05 | 4.93E+05 | 8.52E+05 | 8.86E+05 | 8.35E+05 | 1.22E+00 | 2.10E+00 | 1.07E+00 | up |
| pme0264 | C10H14N2O5 | Thymidine | Nucleotides and derivatives | Nucleotides and derivatives | 50-89-5 | B | 2.79E+05 | 4.07E+05 | 1.83E+05 | 6.70E+04 | 7.88E+04 | 6.90E+04 | 1.19E+00 | 2.47E-01 | -2.02E+00 | down |
| pme2266 | C10H16N2O3S | Biotin | Others | Vitamin | 58-85-5 | B | 1.77E+05 | 1.91E+05 | 2.19E+05 | 9.28E+04 | 9.22E+04 | 9.27E+04 | 1.25E+00 | 4.73E-01 | -1.08E+00 | down |
| Lmhp002031 | C12H24N2O3 | Leu- Leu | Amino acids and derivatives | Amino acids and derivatives | - | A | 4.61E+05 | 4.54E+05 | 5.46E+05 | 9.95E+05 | 1.10E+06 | 1.12E+06 | 1.25E+00 | 2.20E+00 | 1.13E+00 | up |
| pmb2591 | C13H14N2O3 | Acetyltryptophan | Amino acids and derivatives | Amino acids and derivatives | 2280-01-5 | A | 1.33E+05 | 1.12E+05 | 1.21E+05 | 3.41E+05 | 3.38E+05 | 5.15E+05 | 1.23E+00 | 3.26E+00 | 1.71E+00 | up |
| mws1090 | C6H13O9P | Glucose-1-phosphate* | Others | Saccharides and Alcohols | 59-56-3 | B | 3.13E+06 | 2.88E+06 | 3.01E+06 | 1.27E+06 | 1.42E+06 | 1.29E+06 | 1.26E+00 | 4.41E-01 | -1.18E+00 | down |
| mws1297 | C13H18O6 | Benzyl β-D-Glucopyranoside | Phenolic acids | Phenolic acids | 4304-12-5 | A | 1.47E+04 | 1.78E+04 | 1.56E+04 | 8.30E+04 | 8.49E+04 | 9.09E+04 | 1.26E+00 | 5.39E+00 | 2.43E+00 | up |
| Lmmn003323 | C16H32O3 | 2-hydroxyhexadecanoic acid | Organic acids | Organic acids | 764-67-0 | B | 1.15E+06 | 1.04E+06 | 1.29E+06 | 4.45E+06 | 4.44E+06 | 4.63E+06 | 1.26E+00 | 3.88E+00 | 1.96E+00 | up |
| pme1712 | C11H20N2O6 | L-Saccharopine | Amino acids and derivatives | Amino acids and derivatives | 997-68-2 | B | 7.53E+04 | 7.97E+04 | 1.23E+05 | 2.27E+05 | 2.05E+05 | 1.85E+05 | 1.17E+00 | 2.22E+00 | 1.15E+00 | up |
| mws1038 | C11H22N2O4S | (R)-Pantetheine | Others | Others | 496-65-1 | B | 8.06E+04 | 7.50E+04 | 1.00E+05 | 2.81E+05 | 1.94E+05 | 2.87E+05 | 1.22E+00 | 2.98E+00 | 1.57E+00 | up |
| mws5035 | C15H22N2O3 | Leucylphenylalanine | Amino acids and derivatives | Amino acids and derivatives | 56217-82-4 | A | 2.09E+04 | 2.33E+04 | 2.71E+04 | 5.76E+04 | 4.58E+04 | 5.16E+04 | 1.23E+00 | 2.18E+00 | 1.12E+00 | up |
| pmb0889 | C18H30O2 | Punicic acid | Lipids | Free fatty acids | 544-72-9 | B | 1.28E+05 | 1.53E+05 | 2.47E+05 | 4.06E+05 | 4.13E+05 | 4.15E+05 | 1.16E+00 | 2.33E+00 | 1.22E+00 | up |
| Lmhn002926 | C13H12O7 | p-coumaroylmalic acid | Phenolic acids | Phenolic acids | - | B | 2.63E+05 | 2.05E+05 | 2.75E+05 | 9.55E+04 | 1.28E+05 | 1.46E+05 | 1.16E+00 | 4.97E-01 | -1.01E+00 | down |
| Lmhn002051 | C12H12O8 | vnilloylmalic acid | Phenolic acids | Phenolic acids | - | B | 1.01E+04 | 8.88E+03 | 7.14E+03 | 1.06E+05 | 8.52E+04 | 7.36E+04 | 1.26E+00 | 1.02E+01 | 3.34E+00 | up |
| pma3606 | C18H30O3 | 9-Hydroxy-10,12,15-octadecatrienoic acid* | Lipids | Glycerol ester | - | B | 1.33E+05 | 1.56E+05 | 2.11E+05 | 4.95E+05 | 5.67E+05 | 5.88E+05 | 1.23E+00 | 3.30E+00 | 1.72E+00 | up |
| Lmhn102452 | C13H12O8 | Caffeoylmalic acid isomer | Phenolic acids | Phenolic acids | - | B | 8.18E+04 | 6.72E+04 | 1.15E+05 | 2.75E+04 | 4.16E+04 | 4.15E+04 | 1.14E+00 | 4.18E-01 | -1.26E+00 | down |
| pmb2778 | C18H32O3 | 9,10-EODE* | Lipids | Free fatty acids | 65167-83-1 | B | 4.96E+05 | 5.11E+05 | 7.63E+05 | 1.42E+06 | 1.40E+06 | 1.61E+06 | 1.21E+00 | 2.50E+00 | 1.32E+00 | up |
| Rfmb091 | C18H32O3 | 9-Hydroxy-10,12-octadecadienoic acid* | Lipids | Free fatty acids | 15514-85-9 | B | 2.41E+05 | 2.52E+05 | 3.77E+05 | 7.23E+05 | 7.18E+05 | 8.41E+05 | 1.21E+00 | 2.62E+00 | 1.39E+00 | up |
| pmn001610 | C20H36O2 | Eicosadienoic acid | Lipids | Free fatty acids | 5598-38-9 | B | 3.31E+05 | 3.72E+05 | 4.74E+05 | 8.35E+05 | 8.34E+05 | 7.96E+05 | 1.22E+00 | 2.10E+00 | 1.07E+00 | up |
| Lmhn003074 | C14H14O8 | feruloylmalic acid | Phenolic acids | Phenolic acids | - | A | 9.45E+06 | 7.95E+06 | 1.06E+07 | 2.57E+06 | 4.10E+06 | 4.77E+06 | 1.16E+00 | 4.08E-01 | -1.29E+00 | down |
| mws0636 | C18H20N2O3 | Phe-Phe | Amino acids and derivatives | Amino acids and derivatives | 2577-40-4 | A | 2.10E+05 | 1.94E+05 | 2.37E+05 | 5.00E+04 | 4.83E+04 | 6.07E+04 | 1.25E+00 | 2.48E-01 | -2.01E+00 | down |
| pmb2804 | C18H32O4 | 13-HPODE | Lipids | Glycerol ester | 33964-75-9 | B | 1.47E+04 | 1.27E+04 | 1.50E+04 | 3.44E+04 | 3.41E+04 | 3.44E+04 | 1.26E+00 | 2.43E+00 | 1.28E+00 | up |
| Rfmb087 | C18H32O4 | 13-Oxo-9-hydroxy-10-octadecenoic acid* | Lipids | Free fatty acids | - | B | 1.92E+04 | 2.03E+04 | 3.28E+04 | 8.16E+04 | 9.39E+04 | 9.73E+04 | 1.22E+00 | 3.77E+00 | 1.92E+00 | up |
| mws2627 | C16H12O7 | Tamarixetin (3,3',5,7-Tetrahydroxy-4'-Methoxyflavone)* | Flavonoids | Flavonols | 603-61-2 | A | 1.85E+04 | 1.07E+04 | 7.35E+03 | 1.92E+03 | 2.16E+03 | 3.86E+03 | 1.15E+00 | 2.17E-01 | -2.20E+00 | down |
| mws0066 | C16H12O7 | Isorhamnetin* | Flavonoids | Flavonols | 480-19-3 | A | 2.09E+05 | 1.26E+05 | 9.07E+04 | 2.49E+04 | 3.88E+04 | 4.50E+04 | 1.15E+00 | 2.55E-01 | -1.97E+00 | down |
| pme2074 | C18H29NO4 | N-[(-)-Jasmonoyl]-(L)-Isoleucine (JA-L-Ile) | Organic acids | Organic acids | 120330-93-0 | A | 5.78E+05 | 4.18E+05 | 5.79E+05 | 8.65E+04 | 8.29E+04 | 7.12E+04 | 1.26E+00 | 1.53E-01 | -2.71E+00 | down |
| pmf0359 | C15H16O8 | Skimmin | Lignans and Coumarins | Coumarins | 93-39-0 | B | 1.27E+05 | 1.18E+05 | 9.44E+04 | 3.78E+05 | 2.79E+05 | 2.28E+05 | 1.19E+00 | 2.60E+00 | 1.38E+00 | up |
| pmn001419 | C15H18O8 | 1-O-[(E)-p-Cumaroyl]-β-D-glucopyranose* | Phenolic acids | Phenolic acids | - | A | 5.67E+05 | 5.01E+05 | 5.36E+05 | 6.60E+06 | 5.78E+06 | 6.45E+06 | 1.26E+00 | 1.17E+01 | 3.55E+00 | up |
| HmLn002358 | C14H18O9 | Vanillic acid-glucoside | Phenolic acids | Phenolic acids | - | A | 6.87E+05 | 7.41E+05 | 7.01E+05 | 2.48E+06 | 2.18E+06 | 1.69E+06 | 1.24E+00 | 2.98E+00 | 1.58E+00 | up |
| pmn001517 | C15H22O8 | 3,4,5-Trimethoxyphenyl-β-D-Glucopyranoside | Phenolic acids | Phenolic acids | - | A | 2.63E+06 | 2.56E+06 | 2.74E+06 | 6.99E+06 | 6.87E+06 | 5.91E+06 | 1.25E+00 | 2.49E+00 | 1.32E+00 | up |
| Lmfn000604 | C13H16O10 | 6-O-Galloyl-β-D-glucose* | Tannins | Tannin | 13186-19-1 | A | 8.41E+05 | 9.37E+05 | 7.54E+05 | 3.36E+06 | 2.59E+06 | 2.86E+06 | 1.25E+00 | 3.48E+00 | 1.80E+00 | up |
| HmLn000873 | C13H16O10 | 2-O-Galloyl-β-D-glucose | Tannins | Tannin | - | A | 8.22E+05 | 8.97E+05 | 8.06E+05 | 3.27E+06 | 2.85E+06 | 2.73E+06 | 1.26E+00 | 3.51E+00 | 1.81E+00 | up |
| Xmgp006913 | C20H20O5 | 2,4,2',4'-tetrahydroxy-3'-prenylchalcone | Others | Others | - | B | 2.81E+04 | 2.68E+04 | 1.77E+04 | 4.98E+04 | 4.61E+04 | 5.81E+04 | 1.17E+00 | 2.12E+00 | 1.08E+00 | up |
| mws0609 | C10H12N5O7P | Guanosine 3',5'-cyclic monophosphate | Nucleotides and derivatives | Nucleotides and derivatives | 7665-99-8 | A | 7.03E+05 | 7.42E+05 | 7.68E+05 | 2.93E+05 | 3.35E+05 | 2.22E+05 | 1.23E+00 | 3.84E-01 | -1.38E+00 | down |
| mws1077 | C16H18O9 | Scopolin | Lignans and Coumarins | Coumarins | 531-44-2 | A | 1.42E+07 | 1.37E+07 | 1.30E+07 | 4.42E+06 | 4.17E+06 | 3.99E+06 | 1.26E+00 | 3.08E-01 | -1.70E+00 | down |
| Zmhn002422 | C16H20O9 | Feruloyl glucose | Phenolic acids | Phenolic acids | - | A | 5.62E+05 | 6.35E+05 | 6.17E+05 | 1.65E+06 | 1.58E+06 | 1.73E+06 | 1.26E+00 | 2.73E+00 | 1.45E+00 | up |
| Lmgp003270 | C16H16O10 | Scopoletin Beta-D-Glucuronide | Lignans and Coumarins | Coumarins | 132752-65-9 | B | 1.46E+05 | 1.25E+05 | 1.36E+05 | 3.96E+05 | 4.70E+05 | 4.33E+05 | 1.26E+00 | 3.19E+00 | 1.67E+00 | up |
| pmn001695 | C16H20O10 | Trihydroxycinnamoylquinic acid | Phenolic acids | Phenolic acids | - | B | 1.22E+05 | 9.97E+04 | 1.23E+05 | 4.86E+05 | 5.30E+05 | 4.97E+05 | 1.26E+00 | 4.38E+00 | 2.13E+00 | up |
| pme3337 | C14H17N5O8 | N6-Succinyl Adenosine | Nucleotides and derivatives | Nucleotides and derivatives | 4542-23-8 | A | 2.65E+06 | 2.72E+06 | 3.03E+06 | 6.81E+06 | 6.67E+06 | 6.86E+06 | 1.26E+00 | 2.42E+00 | 1.28E+00 | up |
| Zmbn00584 | C12H21NO10S2 | 2-Hydroxy-4-Pentenyl Glucosinolate | Others | Glucosinolates | 19764-03-5 | A | 1.42E+07 | 1.11E+07 | 1.30E+07 | 6.65E+06 | 5.99E+06 | 6.37E+06 | 1.23E+00 | 4.95E-01 | -1.01E+00 | down |
| Lmyn002971 | C13H25NO9S2 | 3-methylpentyl glucosinolate | Others | Glucosinolates | - | A | 1.81E+06 | 1.93E+06 | 1.61E+06 | 7.96E+05 | 8.43E+05 | 9.02E+05 | 1.25E+00 | 4.75E-01 | -1.08E+00 | down |
| pme3007 | C9H14N2O12P2 | Uridine 5'-diphosphate | Nucleotides and derivatives | Nucleotides and derivatives | 27821-45-0 | B | 3.62E+04 | 3.56E+04 | 3.42E+04 | 1.19E+04 | 8.44E+03 | 5.58E+03 | 1.21E+00 | 2.44E-01 | -2.03E+00 | down |
| Lmyn002435 | C19H28O10 | Hydrangeifolin I | Others | Others | - | B | 2.99E+05 | 3.77E+05 | 3.61E+05 | 6.06E+04 | 4.86E+04 | 4.51E+04 | 1.26E+00 | 1.49E-01 | -2.75E+00 | down |
| pme2117 | C10H15N5O10P2 | Adenosine 5'-Diphosphate | Nucleotides and derivatives | Nucleotides and derivatives | 58-64-0 | A | 2.39E+05 | 2.23E+05 | 2.22E+05 | 8.88E+04 | 9.98E+04 | 9.39E+04 | 1.26E+00 | 4.13E-01 | -1.28E+00 | down |
| HJN087 | C21H22O10 | Naringenin-O-glucoside* | Flavonoids | Flavonoid | - | A | 1.02E+06 | 9.63E+05 | 9.51E+05 | 4.37E+06 | 3.69E+06 | 3.34E+06 | 1.26E+00 | 3.89E+00 | 1.96E+00 | up |
| HJN090 | C21H22O10 | Butin-O-glucoside* | Flavonoids | Flavonoid | - | A | 9.42E+05 | 8.76E+05 | 9.81E+05 | 4.03E+06 | 2.92E+06 | 3.25E+06 | 1.25E+00 | 3.65E+00 | 1.87E+00 | up |
| Xmyp005893 | C21H20O11 | Kaempferol-7-O-β-D-glucopyranoside* | Flavonoids | Flavonoid | - | A | 3.33E+05 | 2.67E+05 | 3.88E+05 | 1.46E+05 | 1.29E+05 | 2.01E+05 | 1.14E+00 | 4.82E-01 | -1.05E+00 | down |
| Hmpp003270 | C21H20O11 | Luteolin-4'-O-β-D-glucoside* | Flavonoids | Flavonoid | 6920-38-3 | A | 3.75E+05 | 2.75E+05 | 3.79E+05 | 1.43E+05 | 1.30E+05 | 1.89E+05 | 1.18E+00 | 4.50E-01 | -1.15E+00 | down |
| HJN017 | C21H22O11 | Dihydrokaempferol -O-glucoside* | Flavonoids | Flavonoid | - | B | 1.21E+05 | 1.21E+05 | 7.90E+04 | 2.40E+05 | 2.02E+05 | 2.23E+05 | 1.18E+00 | 2.07E+00 | 1.05E+00 | up |
| Lmtn002796 | C21H22O11 | Aromadendrin 7-glucoside | Terpenoids | Triterpene Saponin | - | B | 3.61E+05 | 2.83E+05 | 2.69E+05 | 1.08E+06 | 1.01E+06 | 9.04E+05 | 1.25E+00 | 3.28E+00 | 1.71E+00 | up |
| mad2085 | C22H18O11 | Caffeoyl-p-coumaroyltartaric acid | Phenolic acids | Phenolic acids | - | B | 9.00E+00 | 9.00E+00 | 9.00E+00 | 4.95E+04 | 4.86E+04 | 4.20E+04 | 1.27E+00 | 5.19E+03 | 1.23E+01 | up |
| Cmyn102345 | C14H27NO10S3 | 6-Methylsulfinylhexyl Glucosinolate | Others | Glucosinolates | 33049-17-1 | B | 1.79E+07 | 1.79E+07 | 2.03E+07 | 8.28E+06 | 9.83E+06 | 9.46E+06 | 1.24E+00 | 4.92E-01 | -1.02E+00 | down |
| pmd0130 | C22H46NO7P | LysoPC 14:0 | Lipids | LPC | - | A | 9.00E+00 | 9.00E+00 | 9.00E+00 | 1.34E+03 | 9.73E+02 | 8.14E+02 | 1.26E+00 | 1.16E+02 | 6.86E+00 | up |
| Lmhn003689 | C23H20O11 | p-coumaroylferuloyltartaric acid | Phenolic acids | Phenolic acids | - | B | 1.35E+04 | 1.04E+04 | 6.42E+03 | 2.23E+04 | 1.91E+04 | 2.10E+04 | 1.10E+00 | 2.06E+00 | 1.04E+00 | up |
| Lmzn006284 | C30H48O4 | 2α-hydroxyursolic acid | Terpenoids | Triterpene | - | A | 9.00E+00 | 9.00E+00 | 9.00E+00 | 1.58E+05 | 2.09E+05 | 1.82E+05 | 1.27E+00 | 2.03E+04 | 1.43E+01 | up |
| Lmmn003398 | C23H22O12 | Kaempferol acetyl-glucoside | Flavonoids | Flavonols | - | B | 2.93E+04 | 1.60E+04 | 2.48E+04 | 1.46E+05 | 1.14E+05 | 1.30E+05 | 1.24E+00 | 5.56E+00 | 2.48E+00 | up |
| HJAP135 | C24H24O12 | Dihydrochrysin-O-malonylglucoside | Flavonoids | Flavonoid | - | A | 2.64E+05 | 2.83E+05 | 1.62E+05 | 2.16E+06 | 1.49E+06 | 1.58E+06 | 1.24E+00 | 7.37E+00 | 2.88E+00 | up |
| Hmln002199 | C23H22O13 | Quercetin-3-O-(6''-O-acetyl)-galactoside | Flavonoids | Flavonols | - | B | 9.07E+03 | 4.53E+03 | 6.05E+03 | 3.63E+04 | 2.17E+04 | 3.25E+04 | 1.20E+00 | 4.60E+00 | 2.20E+00 | up |
| pmn001644 | C23H20O14 | Quercetin-3-O-(2''-acetyl)-β-D-glucuronide | Flavonoids | Flavonols | - | B | 4.61E+04 | 2.55E+04 | 4.65E+04 | 1.26E+05 | 1.23E+05 | 1.04E+05 | 1.19E+00 | 2.99E+00 | 1.58E+00 | up |
| pmb3013 | C24H24O13 | Isorhamnetin-O-acetyl-hexoside* | Flavonoids | Flavonols | - | B | 1.56E+05 | 1.56E+05 | 1.47E+05 | 5.33E+05 | 4.17E+05 | 4.99E+05 | 1.26E+00 | 3.15E+00 | 1.66E+00 | up |
| Lmhn003801 | C25H24O13 | feruloylsinapoyltartaric acid* | Phenolic acids | Phenolic acids | - | B | 1.52E+04 | 1.43E+04 | 3.37E+04 | 5.88E+04 | 7.27E+04 | 7.20E+04 | 1.15E+00 | 3.22E+00 | 1.69E+00 | up |
| Hmln002402 | C24H22O14 | Kaempferol-3-O-(6''-malonyl)-galactoside* | Flavonoids | Flavonols | - | B | 9.00E+00 | 9.00E+00 | 1.30E+04 | 6.40E+04 | 4.52E+04 | 6.58E+04 | 1.00E+00 | 1.35E+01 | 3.75E+00 | up |
| HJAP065 | C24H22O14 | Kaempferol-malonyl-3-O-glucoside* | Flavonoids | Flavonoid | - | A | 2.68E+04 | 1.48E+04 | 2.27E+04 | 1.42E+05 | 8.81E+04 | 1.19E+05 | 1.23E+00 | 5.43E+00 | 2.44E+00 | up |
| pmb0824 | C25H28O13 | Syringic acid O-feruloyl-O-hexoside | Phenolic acids | Phenolic acids | - | B | 1.28E+05 | 1.26E+05 | 1.25E+05 | 3.25E+05 | 3.03E+05 | 2.72E+05 | 1.26E+00 | 2.37E+00 | 1.24E+00 | up |
| pmb0608 | C25H24O14 | Chrysoeriol-O-malonylhexoside | Flavonoids | Flavonoid | - | B | 9.42E+03 | 9.60E+03 | 6.17E+03 | 1.75E+04 | 2.03E+04 | 1.96E+04 | 1.19E+00 | 2.28E+00 | 1.19E+00 | up |
| pmn001672 | C23H32O15 | β-D-Furanofructosyl-α-D-(3-mustard acyl)glucoside* | Phenolic acids | Phenolic acids | - | B | 4.10E+05 | 5.02E+05 | 8.41E+05 | 2.44E+05 | 3.10E+05 | 2.35E+05 | 1.09E+00 | 4.50E-01 | -1.15E+00 | down |
| pmp000589 | C24H22O15 | Quercetin-7-O-(6'-O-malonyl)-β-D-glucoside* | Flavonoids | Flavonols | - | B | 8.33E+03 | 6.91E+03 | 1.01E+04 | 2.74E+04 | 1.96E+04 | 2.08E+04 | 1.21E+00 | 2.67E+00 | 1.42E+00 | up |
| HJAP064 | C25H24O15 | Isorhamnetin O-malonylglucoside* | Flavonoids | Flavonoid | - | B | 2.26E+05 | 2.19E+05 | 1.97E+05 | 5.95E+05 | 4.52E+05 | 6.86E+05 | 1.23E+00 | 2.70E+00 | 1.43E+00 | up |
| Zmhp005139 | C25H24O15 | Tamarixetin-malonylhexoside* | Flavonoids | Flavonoid | - | A | 2.46E+05 | 2.09E+05 | 2.08E+05 | 6.33E+05 | 4.74E+05 | 7.02E+05 | 1.23E+00 | 2.73E+00 | 1.45E+00 | up |
| pmb2819 | C27H38O19 | O-Caffeoyl maltotriose | Phenolic acids | Phenolic acids | - | B | 4.99E+03 | 4.85E+03 | 7.42E+03 | 2.24E+04 | 2.46E+04 | 1.82E+04 | 1.23E+00 | 3.78E+00 | 1.92E+00 | up |
| Lmjn003562 | C32H38O17 | 3,6'-O-diferuloylsucrose | Phenolic acids | Phenolic acids | 107172-40-7 | A | 5.51E+06 | 5.89E+06 | 6.59E+06 | 1.45E+06 | 1.53E+06 | 1.36E+06 | 1.26E+00 | 2.41E-01 | -2.05E+00 | down |
| pmb0709 | C30H32O20 | Quercetin-7-O-malonylhexosyl-hexoside | Flavonoids | Flavonols | - | B | 3.43E+04 | 2.77E+04 | 2.08E+04 | 5.60E+04 | 5.51E+04 | 5.80E+04 | 1.18E+00 | 2.04E+00 | 1.03E+00 | up |
| Lmdp004696 | C38H40O20 | Kaempferol-O-sinapoylglucosid-O-glucoside | Flavonoids | Flavonols | - | A | 2.19E+04 | 2.66E+04 | 1.77E+04 | 7.53E+03 | 5.59E+03 | 1.34E+04 | 1.09E+00 | 4.02E-01 | -1.32E+00 | down |
| Lmdp004426 | C38H40O21 | Quercetin-O-sinapoylglucosid-O-glucoside | Flavonoids | Flavonols | - | B | 2.99E+04 | 3.15E+04 | 2.54E+04 | 1.16E+04 | 1.14E+04 | 2.03E+04 | 1.10E+00 | 4.99E-01 | -1.00E+00 | down |

**Supplemental Table 2 Differential metabolites between NSD and SSD in the maintainer line.**

| Index | Formula | Compounds | Class I | Class II | CAS | Level | B-BM1 | B-BM2 | B-BM3 | B-AM1 | B-AM2 | B-AM3 | VIP | Fold_Change | Log2FC | Type |
| --- | --- | --- | --- | --- | --- | --- | --- | --- | --- | --- | --- | --- | --- | --- | --- | --- |
| mws0576 | C4H8O3 | 3-Hydroxybutyrate* | Organic acids | Organic acids | 300-85-6 | A | 1.25E+06 | 1.97E+06 | 1.26E+06 | 9.00E+00 | 9.00E+00 | 9.00E+00 | 1.20E+00 | 6.02E-06 | -1.73E+01 | down |
| pmb1096 | C8H7N | Indole | Alkaloids | Plumerane | 120-72-9 | B | 7.66E+04 | 6.77E+04 | 8.78E+04 | 3.26E+04 | 2.95E+04 | 2.89E+04 | 1.18E+00 | 3.92E-01 | -1.35E+00 | down |
| pmp001287 | C8H9N | N-Benzylmethylene isomethylamine | Alkaloids | Alkaloids | - | A | 9.02E+06 | 8.19E+06 | 1.03E+07 | 3.66E+06 | 3.70E+06 | 3.72E+06 | 1.18E+00 | 4.03E-01 | -1.31E+00 | down |
| mws0251 | C5H6N2O2 | Thymine | Nucleotides and derivatives | Nucleotides and derivatives | 65-71-4 | A | 2.84E+04 | 3.52E+04 | 2.67E+04 | 5.20E+03 | 5.53E+03 | 6.53E+03 | 1.18E+00 | 1.91E-01 | -2.39E+00 | down |
| pme0193 | C5H10N2O3 | L-Glutamine | Amino acids and derivatives | Amino acids and derivatives | 56-85-9 | A | 8.97E+06 | 7.23E+06 | 9.14E+06 | 3.96E+06 | 3.70E+06 | 4.22E+06 | 1.17E+00 | 4.69E-01 | -1.09E+00 | down |
| pme0026 | C6H14N2O2 | L-(+)-Lysine | Amino acids and derivatives | Amino acids and derivatives | 56-87-1 | A | 1.03E+07 | 8.36E+06 | 1.04E+07 | 4.50E+06 | 4.53E+06 | 4.95E+06 | 1.17E+00 | 4.81E-01 | -1.06E+00 | down |
| mws1050 | C5H9NO4 | O-Acetylserine* | Amino acids and derivatives | Amino acids and derivatives | 5147-00-2 | A | 7.24E+05 | 7.04E+05 | 7.91E+05 | 2.56E+05 | 2.38E+05 | 2.39E+05 | 1.19E+00 | 3.30E-01 | -1.60E+00 | down |
| pme0256 | C5H4N4O2 | Xanthine | Nucleotides and derivatives | Nucleotides and derivatives | 69-89-6 | B | 1.05E+05 | 9.09E+04 | 1.28E+05 | 3.90E+04 | 2.83E+04 | 6.19E+04 | 1.06E+00 | 3.99E-01 | -1.33E+00 | down |
| mws0254 | C6H9N3O2 | L-Histidine | Amino acids and derivatives | Amino acids and derivatives | 71-00-1 | A | 4.64E+06 | 4.78E+06 | 4.41E+06 | 2.21E+06 | 1.55E+06 | 2.07E+06 | 1.16E+00 | 4.22E-01 | -1.24E+00 | down |
| mws1346 | C6H11NO4 | DL-2-Aminoadipic acid | Alkaloids | Alkaloids | 542-32-5 | B | 2.20E+06 | 1.91E+06 | 2.08E+06 | 8.51E+05 | 8.71E+05 | 9.36E+05 | 1.19E+00 | 4.29E-01 | -1.22E+00 | down |
| mws1320 | C10H11NO | Tryptophol | Alkaloids | Plumerane | 526-55-6 | B | 1.04E+05 | 1.09E+05 | 1.02E+05 | 4.62E+04 | 3.33E+04 | 4.37E+04 | 1.17E+00 | 3.91E-01 | -1.35E+00 | down |
| pme3968 | C6H7N5O | 7-Methylguanine | Nucleotides and derivatives | Nucleotides and derivatives | 578-76-7 | A | 5.22E+05 | 4.21E+05 | 6.41E+05 | 1.95E+05 | 1.86E+05 | 1.79E+05 | 1.16E+00 | 3.53E-01 | -1.50E+00 | down |
| pme0021 | C9H11NO2 | L-Phenylalanine | Amino acids and derivatives | Amino acids and derivatives | 63-91-2 | A | 3.19E+07 | 2.71E+07 | 3.61E+07 | 1.27E+07 | 1.33E+07 | 1.26E+07 | 1.17E+00 | 4.05E-01 | -1.30E+00 | down |
| pmc0274 | C6H6N4S | 6-Methylmercaptopurine | Nucleotides and derivatives | Nucleotides and derivatives | 50-66-8 | B | 3.11E+07 | 2.90E+07 | 3.60E+07 | 1.43E+07 | 1.44E+07 | 1.41E+07 | 1.18E+00 | 4.45E-01 | -1.17E+00 | down |
| mws0260 | C6H14N4O2 | L-(+)-Arginine | Amino acids and derivatives | Amino acids and derivatives | 74-79-3 | A | 6.76E+06 | 5.25E+06 | 7.41E+06 | 1.14E+06 | 7.58E+05 | 1.13E+06 | 1.18E+00 | 1.56E-01 | -2.68E+00 | down |
| pmf0367 | C9H6O4 | 5,7-Dihydroxychromone | Others | Others | 31721-94-5 | B | 1.58E+04 | 1.71E+04 | 2.18E+04 | 1.02E+04 | 6.95E+03 | 7.83E+03 | 1.12E+00 | 4.57E-01 | -1.13E+00 | down |
| pme2596 | C8H9NO4 | 4-Pyridoxic acid | Others | Vitamin | 82-82-6 | B | 3.67E+05 | 3.41E+05 | 4.38E+05 | 1.29E+05 | 1.36E+05 | 1.44E+05 | 1.18E+00 | 3.58E-01 | -1.48E+00 | down |
| Hmmp001310 | C11H9NO2 | 3-Indoleacrylic acid | Alkaloids | Alkaloids | 1204-06-4 | A | 4.29E+07 | 3.63E+07 | 4.90E+07 | 1.66E+07 | 1.82E+07 | 1.85E+07 | 1.17E+00 | 4.15E-01 | -1.27E+00 | down |
| pme3388 | C7H16N4O2 | H-HomoArg-OH | Amino acids and derivatives | Amino acids and derivatives | 156-86-5 | B | 1.99E+05 | 2.00E+05 | 2.75E+05 | 7.62E+04 | 7.39E+04 | 7.54E+04 | 1.17E+00 | 3.34E-01 | -1.58E+00 | down |
| pme0278 | C7H14N2O4 | 2,6-Diaminooimelic acid | Amino acids and derivatives | Amino acids and derivatives | 583-93-7 | B | 1.57E+05 | 1.70E+05 | 1.89E+05 | 8.75E+04 | 7.01E+04 | 8.00E+04 | 1.17E+00 | 4.60E-01 | -1.12E+00 | down |
| Hmcp002123 | C10H8O4 | 6-Hydroxy-7-methoxycoumarin | Lignans and Coumarins | Coumarins | 776-86-3 | A | 5.54E+05 | 7.19E+05 | 6.11E+05 | 5.88E+04 | 6.01E+04 | 6.07E+04 | 1.19E+00 | 9.54E-02 | -3.39E+00 | down |
| pme3705 | C6H10O7 | D-Glucoronic acid* | Others | Saccharides and Alcohols | 6556-12-3 | B | 1.85E+05 | 1.96E+05 | 1.61E+05 | 3.91E+04 | 3.11E+04 | 3.90E+04 | 1.19E+00 | 2.02E-01 | -2.31E+00 | down |
| mws1189 | C6H10O7 | D-Galacturonic acid(Gal A)* | Organic acids | Organic acids | 685-73-4 | A | 1.65E+05 | 2.61E+05 | 1.61E+05 | 5.41E+04 | 4.20E+04 | 5.24E+04 | 1.16E+00 | 2.53E-01 | -1.99E+00 | down |
| mws0282 | C11H12N2O2 | L-Tryptophan | Amino acids and derivatives | Amino acids and derivatives | 73-22-3 | A | 2.79E+07 | 2.34E+07 | 3.60E+07 | 7.16E+06 | 8.36E+06 | 8.67E+06 | 1.17E+00 | 2.77E-01 | -1.85E+00 | down |
| pmb0818 | C11H11NO3 | Methoxyindoleacetic acid | Alkaloids | Plumerane | 3471-31-6 | B | 1.67E+06 | 1.29E+06 | 2.03E+06 | 4.81E+05 | 5.11E+05 | 5.21E+05 | 1.17E+00 | 3.03E-01 | -1.72E+00 | down |
| mws1212 | C11H12O4 | Methyl ferulate | Phenolic acids | Phenolic acids | 2309-07-1 | A | 5.81E+06 | 6.53E+06 | 5.15E+06 | 1.77E+06 | 1.82E+06 | 1.60E+06 | 1.19E+00 | 2.97E-01 | -1.75E+00 | down |
| pmb2620 | C11H12O4 | 3,4-Dimethoxycinnamic acid | Phenolic acids | Phenolic acids | 14737-89-4 | A | 5.94E+06 | 6.49E+06 | 5.01E+06 | 2.17E+06 | 2.00E+06 | 1.87E+06 | 1.18E+00 | 3.47E-01 | -1.53E+00 | down |
| mws0853 | C11H14O4 | Sinapyl alcohol | Phenolic acids | Phenolic acids | 537-33-7 | B | 3.23E+04 | 2.50E+04 | 2.13E+04 | 9.92E+03 | 1.24E+04 | 1.64E+04 | 1.05E+00 | 4.93E-01 | -1.02E+00 | down |
| pmb2507 | C5H11O7P | 2-Deoxyribose 1-phosphate | Others | Others | 17210-42-3 | B | 2.26E+05 | 1.75E+05 | 2.38E+05 | 1.10E+05 | 7.61E+04 | 1.11E+05 | 1.11E+00 | 4.66E-01 | -1.10E+00 | down |
| mws4174 | C8H15NO6 | N-Acetyl-β-D-mannosamine* | Others | Others | 7772-94-3 | B | 1.61E+05 | 1.29E+05 | 2.72E+05 | 5.76E+04 | 7.01E+04 | 4.33E+04 | 1.09E+00 | 3.04E-01 | -1.72E+00 | down |
| mws5042 | C11H14N2O3 | Glycylphenylalanine | Amino acids and derivatives | Amino acids and derivatives | 721-66-4 | A | 3.55E+05 | 3.13E+05 | 3.19E+05 | 1.33E+05 | 1.01E+05 | 1.59E+05 | 1.15E+00 | 3.98E-01 | -1.33E+00 | down |
| pme1194 | C9H13N3O4 | Deoxycytidine | Nucleotides and derivatives | Nucleotides and derivatives | 951-77-9 | B | 9.89E+04 | 9.32E+04 | 1.08E+05 | 3.77E+04 | 3.12E+04 | 4.48E+04 | 1.16E+00 | 3.79E-01 | -1.40E+00 | down |
| pme3882 | C9H12N2O5 | 2'-Deoxyuridine | Nucleotides and derivatives | Nucleotides and derivatives | 951-78-0 | B | 2.75E+04 | 2.74E+04 | 3.37E+04 | 6.40E+03 | 5.73E+03 | 9.33E+03 | 1.16E+00 | 2.42E-01 | -2.04E+00 | down |
| mws0021 | C14H12O3 | Resveratrol | Others | Stilbene | 501-36-0 | B | 4.47E+04 | 5.83E+04 | 4.57E+04 | 2.49E+03 | 2.60E+03 | 2.10E+03 | 1.19E+00 | 4.84E-02 | -4.37E+00 | down |
| Lmhp001461 | C11H20N2O3 | Pro-Leu | Amino acids and derivatives | Amino acids and derivatives | - | A | 4.20E+05 | 3.10E+05 | 4.22E+05 | 1.69E+05 | 1.49E+05 | 2.08E+05 | 1.12E+00 | 4.56E-01 | -1.13E+00 | down |
| pmb0962 | C10H22N2O4 | Lysine butyrate | Amino acids and derivatives | Amino acids and derivatives | 80407-71-2 | B | 2.10E+05 | 1.50E+05 | 2.48E+05 | 8.16E+04 | 9.37E+04 | 9.64E+04 | 1.11E+00 | 4.47E-01 | -1.16E+00 | down |
| pme0264 | C10H14N2O5 | Thymidine | Nucleotides and derivatives | Nucleotides and derivatives | 50-89-5 | B | 2.78E+05 | 2.82E+05 | 3.13E+05 | 7.99E+04 | 7.06E+04 | 8.35E+04 | 1.19E+00 | 2.68E-01 | -1.90E+00 | down |
| pme2266 | C10H16N2O3S | Biotin | Others | Vitamin | 58-85-5 | B | 6.11E+04 | 6.64E+04 | 9.62E+04 | 2.63E+04 | 2.74E+04 | 3.80E+04 | 1.10E+00 | 4.10E-01 | -1.29E+00 | down |
| pme3961 | C10H13N5O3 | Deoxyadenosine* | Nucleotides and derivatives | Nucleotides and derivatives | 958-09-8 | A | 3.99E+06 | 3.32E+06 | 4.03E+06 | 4.98E+05 | 4.49E+05 | 5.12E+05 | 1.19E+00 | 1.29E-01 | -2.96E+00 | down |
| mws1715 | C10H13N5O3 | Cordycepin* | Nucleotides and derivatives | Nucleotides and derivatives | 73-03-0 | B | 5.23E+04 | 4.40E+04 | 6.10E+04 | 6.44E+03 | 8.19E+03 | 8.96E+03 | 1.18E+00 | 1.50E-01 | -2.74E+00 | down |
| Lmhn002629 | C11H10O7 | benzoyltartaric acid | Phenolic acids | Phenolic acids | - | B | 1.67E+04 | 1.76E+04 | 2.00E+04 | 6.51E+03 | 8.28E+03 | 8.16E+03 | 1.17E+00 | 4.23E-01 | -1.24E+00 | down |
| pme1184 | C10H13N5O4 | Deoxyguanosine* | Nucleotides and derivatives | Nucleotides and derivatives | 961-07-9 | B | 1.15E+06 | 1.09E+06 | 1.17E+06 | 1.33E+05 | 1.39E+05 | 1.63E+05 | 1.19E+00 | 1.28E-01 | -2.97E+00 | down |
| pme0376 | C15H12O5 | Naringenin* | Flavonoids | Dihydroflavone | 480-41-1 | A | 7.17E+06 | 7.60E+06 | 8.55E+06 | 3.52E+05 | 4.05E+05 | 2.83E+05 | 1.19E+00 | 4.46E-02 | -4.49E+00 | down |
| pme2960 | C15H12O5 | Naringenin chalcone* | Flavonoids | Chalcones | 73692-50-9 | A | 2.57E+07 | 3.02E+07 | 3.10E+07 | 1.97E+06 | 2.04E+06 | 1.48E+06 | 1.19E+00 | 6.31E-02 | -3.99E+00 | down |
| mws0914 | C15H12O5 | Pinobanksin* | Flavonoids | Dihydroflavonol | 548-82-3 | A | 7.35E+06 | 8.06E+06 | 8.82E+06 | 3.67E+05 | 3.80E+05 | 2.80E+05 | 1.19E+00 | 4.24E-02 | -4.56E+00 | down |
| Lmmn003323 | C16H32O3 | 2-hydroxyhexadecanoic acid | Organic acids | Organic acids | 764-67-0 | B | 1.19E+07 | 7.82E+06 | 1.02E+07 | 3.08E+06 | 3.06E+06 | 2.84E+06 | 1.17E+00 | 3.01E-01 | -1.73E+00 | down |
| pme1201 | C15H14O5 | Phloretin | Flavonoids | Chalcones | 60-82-2 | A | 2.80E+06 | 2.79E+06 | 2.48E+06 | 1.68E+05 | 1.80E+05 | 1.29E+05 | 1.19E+00 | 5.92E-02 | -4.08E+00 | down |
| Lmhn002926 | C13H12O7 | p-coumaroylmalic acid | Phenolic acids | Phenolic acids | - | B | 6.28E+05 | 5.53E+05 | 6.48E+05 | 3.21E+05 | 1.27E+05 | 3.12E+05 | 1.01E+00 | 4.15E-01 | -1.27E+00 | down |
| mws0629 | C13H16N2O5 | Asp-phe | Amino acids and derivatives | Amino acids and derivatives | 13433-09-5 | B | 1.49E+04 | 1.72E+04 | 2.02E+04 | 1.11E+04 | 7.56E+03 | 7.17E+03 | 1.09E+00 | 4.94E-01 | -1.02E+00 | down |
| mws1068 | C15H10O6 | Kaempferol | Flavonoids | Flavonols | 520-18-3 | A | 6.82E+05 | 4.95E+05 | 8.32E+05 | 3.37E+04 | 2.77E+04 | 2.24E+04 | 1.19E+00 | 4.17E-02 | -4.58E+00 | down |
| mws0064 | C15H12O6 | Eriodictyol | Flavonoids | Dihydroflavone | 552-58-9 | A | 1.28E+05 | 1.46E+05 | 1.43E+05 | 1.82E+04 | 2.23E+04 | 1.45E+04 | 1.19E+00 | 1.32E-01 | -2.92E+00 | down |
| mws1094 | C15H12O6 | Dihydrokaempferol | Flavonoids | Dihydroflavonol | 480-20-6 | A | 4.58E+05 | 6.93E+05 | 3.91E+05 | 3.79E+04 | 4.13E+04 | 3.23E+04 | 1.18E+00 | 7.23E-02 | -3.79E+00 | down |
| pme2954 | C15H10O7 | Quercetin | Flavonoids | Flavonols | 117-39-5 | B | 1.00E+05 | 7.70E+04 | 1.61E+05 | 9.00E+00 | 9.00E+00 | 9.00E+00 | 1.19E+00 | 7.98E-05 | -1.36E+01 | down |
| Hmyp002315 | C15H10O7 | 3,5,7,4'--Tetrahydroxy-Coumaronochromone | Others | Others | - | B | 1.61E+05 | 1.15E+05 | 1.47E+05 | 2.81E+04 | 4.14E+04 | 2.84E+04 | 1.17E+00 | 2.32E-01 | -2.11E+00 | down |
| Lmhn003074 | C14H14O8 | feruloylmalic acid | Phenolic acids | Phenolic acids | - | A | 1.35E+07 | 1.38E+07 | 1.37E+07 | 6.79E+06 | 3.32E+06 | 6.29E+06 | 1.08E+00 | 4.00E-01 | -1.32E+00 | down |
| mws0636 | C18H20N2O3 | Phe-Phe | Amino acids and derivatives | Amino acids and derivatives | 2577-40-4 | A | 1.30E+05 | 1.52E+05 | 1.75E+05 | 2.32E+04 | 1.69E+04 | 2.56E+04 | 1.18E+00 | 1.44E-01 | -2.80E+00 | down |
| pmb2804 | C18H32O4 | 13-HPODE | Lipids | Glycerol ester | 33964-75-9 | B | 1.79E+04 | 1.53E+04 | 1.89E+04 | 8.33E+03 | 8.38E+03 | 8.70E+03 | 1.18E+00 | 4.88E-01 | -1.04E+00 | down |
| Rfmb087 | C18H32O4 | 13-Oxo-9-hydroxy-10-octadecenoic acid* | Lipids | Free fatty acids | - | B | 7.00E+04 | 2.94E+04 | 4.67E+04 | 9.13E+03 | 9.06E+03 | 1.19E+04 | 1.13E+00 | 2.06E-01 | -2.28E+00 | down |
| mws2627 | C16H12O7 | Tamarixetin (3,3',5,7-Tetrahydroxy-4'-Methoxyflavone)* | Flavonoids | Flavonols | 603-61-2 | A | 1.15E+05 | 8.00E+04 | 1.61E+05 | 5.82E+03 | 4.05E+03 | 3.99E+03 | 1.18E+00 | 3.90E-02 | -4.68E+00 | down |
| mws0066 | C16H12O7 | Isorhamnetin* | Flavonoids | Flavonols | 480-19-3 | A | 1.32E+06 | 8.97E+05 | 1.77E+06 | 7.45E+04 | 6.14E+04 | 4.95E+04 | 1.18E+00 | 4.65E-02 | -4.43E+00 | down |
| Lmdp003808 | C16H12O7 | Azaleatin (5-O-Methylquercetin) | Flavonoids | Flavonols | 529-51-1 | B | 8.08E+05 | 9.88E+05 | 1.17E+06 | 5.06E+05 | 3.47E+05 | 3.49E+05 | 1.13E+00 | 4.06E-01 | -1.30E+00 | down |
| mws0582 | C11H19N3O6S | S-(methyl)glutathione | Amino acids and derivatives | Amino acids and derivatives | 2922-56-7 | A | 7.92E+04 | 6.74E+04 | 1.10E+05 | 2.71E+04 | 1.76E+04 | 3.75E+04 | 1.09E+00 | 3.20E-01 | -1.64E+00 | down |
| mws0983 | C20H39NO2 | N-Oleoylethanolamine | Others | Others | 111-58-0 | B | 5.64E+05 | 3.48E+05 | 4.55E+05 | 5.83E+04 | 7.46E+04 | 5.28E+04 | 1.18E+00 | 1.36E-01 | -2.88E+00 | down |
| pmn001691 | C18H32O5 | 9,12,13-Trihyroxy-10,15-octadecadienoic acid | Lipids | Free fatty acids | - | A | 3.44E+05 | 3.30E+05 | 2.88E+05 | 3.93E+04 | 4.22E+04 | 3.25E+04 | 1.19E+00 | 1.19E-01 | -3.08E+00 | down |
| pmn001694 | C18H34O5 | 9,10,13-Trihyroxy-11-octadecadienoic acid | Lipids | Free fatty acids | - | B | 9.52E+05 | 8.53E+05 | 7.56E+05 | 5.49E+04 | 6.11E+04 | 4.98E+04 | 1.19E+00 | 6.47E-02 | -3.95E+00 | down |
| mws0675 | C11H15N2O8P | β-Nicotinamide mononucleotide | Nucleotides and derivatives | Nucleotides and derivatives | 1094-61-7 | B | 9.67E+04 | 5.23E+04 | 7.23E+04 | 1.81E+04 | 1.67E+04 | 1.64E+04 | 1.16E+00 | 2.31E-01 | -2.11E+00 | down |
| mws1015 | C15H16O9 | Esculin(6,7-DihydroxyCoumarin-6-glucoside)* | Lignans and Coumarins | Coumarins | 531-75-9 | A | 2.33E+06 | 2.67E+06 | 2.43E+06 | 5.66E+05 | 3.30E+05 | 5.72E+05 | 1.17E+00 | 1.98E-01 | -2.34E+00 | down |
| mws0609 | C10H12N5O7P | Guanosine 3',5'-cyclic monophosphate | Nucleotides and derivatives | Nucleotides and derivatives | 7665-99-8 | A | 7.19E+05 | 7.66E+05 | 7.82E+05 | 2.32E+05 | 2.29E+05 | 2.22E+05 | 1.19E+00 | 3.01E-01 | -1.73E+00 | down |
| pmb0801 | C14H19NO9 | 4-Pyridoxic acid O-hexoside | Others | Vitamin | - | B | 3.36E+04 | 3.04E+04 | 3.73E+04 | 1.51E+04 | 1.45E+04 | 1.53E+04 | 1.18E+00 | 4.42E-01 | -1.18E+00 | down |
| pmb0981 | C10H14N5O7P | Adenosine 5'-monophosphate | Nucleotides and derivatives | Nucleotides and derivatives | 61-19-8 | A | 5.85E+05 | 5.38E+05 | 4.99E+05 | 2.39E+05 | 1.99E+05 | 2.03E+05 | 1.18E+00 | 3.95E-01 | -1.34E+00 | down |
| mws1077 | C16H18O9 | Scopolin | Lignans and Coumarins | Coumarins | 531-44-2 | A | 1.44E+07 | 1.50E+07 | 1.42E+07 | 2.70E+06 | 3.09E+06 | 2.56E+06 | 1.19E+00 | 1.91E-01 | -2.39E+00 | down |
| mws0757 | C10H17NO9S2 | Sinigrin hydrate | Others | Glucosinolates | 3952-98-5 | B | 1.20E+05 | 9.62E+04 | 1.31E+05 | 4.80E+04 | 6.22E+04 | 6.14E+04 | 1.12E+00 | 4.94E-01 | -1.02E+00 | down |
| mws0232 | C17H20N4O6 | Riboflavin | Others | Vitamin | 83-88-5 | B | 2.76E+06 | 2.50E+06 | 2.64E+06 | 4.35E+05 | 4.15E+05 | 4.97E+05 | 1.19E+00 | 1.71E-01 | -2.55E+00 | down |
| pme3337 | C14H17N5O8 | N6-Succinyl Adenosine | Nucleotides and derivatives | Nucleotides and derivatives | 4542-23-8 | A | 2.08E+06 | 1.78E+06 | 2.80E+06 | 4.78E+05 | 4.66E+05 | 4.59E+05 | 1.18E+00 | 2.11E-01 | -2.25E+00 | down |
| pmn001399 | C20H22O9 | 2,4,6,4'-Tetrahydroxy-stilbene-2-O-D-glucopyranoside | Others | Stilbene | - | B | 4.29E+05 | 3.96E+05 | 4.92E+05 | 7.30E+04 | 6.79E+04 | 7.48E+04 | 1.19E+00 | 1.64E-01 | -2.61E+00 | down |
| Lmbp003668 | C20H18O10 | Kaempferol-3-arabinopyranoside | Flavonoids | Flavonoid | - | B | 1.67E+05 | 1.53E+05 | 2.01E+05 | 2.15E+04 | 2.08E+04 | 2.21E+04 | 1.19E+00 | 1.24E-01 | -3.02E+00 | down |
| Lmnn002262 | C13H24NO9S3- | 5-(Methylthio)pentyl thioglucoside （Glucoberteroin） | Others | Glucosinolates | - | A | 4.74E+05 | 4.77E+05 | 6.00E+05 | 2.49E+05 | 2.59E+05 | 2.55E+05 | 1.17E+00 | 4.92E-01 | -1.02E+00 | down |
| mws4183 | C20H18O11 | Quercetin-3-O-α-L-arabinopyranoside(guaijaverin)* | Flavonoids | Flavonols | 22255-13-6 | B | 8.28E+05 | 5.67E+05 | 9.58E+05 | 9.05E+04 | 9.10E+04 | 9.86E+04 | 1.18E+00 | 1.19E-01 | -3.07E+00 | down |
| Zmln001187 | C16H23NO9S2 | 3-Phenylpropyl Glucosinolate | Others | Glucosinolates | 499-27-4 | A | 4.88E+04 | 4.40E+04 | 6.93E+04 | 2.00E+04 | 2.84E+04 | 2.44E+04 | 1.09E+00 | 4.49E-01 | -1.16E+00 | down |
| Xmyp005893 | C21H20O11 | Kaempferol-7-O-β-D-glucopyranoside* | Flavonoids | Flavonoid | - | A | 6.42E+06 | 6.09E+06 | 6.32E+06 | 1.49E+06 | 1.45E+06 | 1.78E+06 | 1.19E+00 | 2.51E-01 | -1.99E+00 | down |
| Hmpp003270 | C21H20O11 | Luteolin-4'-O-β-D-glucoside* | Flavonoids | Flavonoid | 6920-38-3 | A | 6.70E+06 | 6.42E+06 | 6.67E+06 | 1.55E+06 | 1.56E+06 | 1.67E+06 | 1.19E+00 | 2.42E-01 | -2.05E+00 | down |
| mws0061 | C21H20O12 | Quercetin-3-O-β-D-Galactoside (Hyperin)* | Flavonoids | Flavonols | 482-36-0 | B | 2.52E+06 | 1.81E+06 | 1.61E+06 | 6.10E+05 | 5.94E+05 | 5.04E+05 | 1.17E+00 | 2.88E-01 | -1.80E+00 | down |
| mws1329 | C21H20O12 | Quercetin-7-O-Glucoside* | Flavonoids | Flavonols | 491-50-9 | A | 2.05E+07 | 1.83E+07 | 1.82E+07 | 6.44E+06 | 6.19E+06 | 6.20E+06 | 1.19E+00 | 3.31E-01 | -1.60E+00 | down |
| mws0091 | C21H20O12 | Quercetin-3-O-β-D-glucoside(Isoquercitrin)* | Flavonoids | Flavonols | 482-35-9 | A | 3.11E+07 | 3.09E+07 | 2.72E+07 | 1.04E+07 | 1.07E+07 | 8.60E+06 | 1.18E+00 | 3.33E-01 | -1.59E+00 | down |
| pme1598 | C22H24O11 | Hesperetin 5-O-glucoside | Flavonoids | Dihydroflavonol | 69651-80-5 | B | 2.95E+07 | 2.51E+07 | 2.28E+07 | 9.41E+06 | 8.51E+06 | 7.91E+06 | 1.18E+00 | 3.34E-01 | -1.58E+00 | down |
| Lmhn003689 | C23H20O11 | p-coumaroylferuloyltartaric acid | Phenolic acids | Phenolic acids | - | B | 6.44E+04 | 5.34E+04 | 4.51E+04 | 2.12E+04 | 1.96E+04 | 2.44E+04 | 1.16E+00 | 4.01E-01 | -1.32E+00 | down |
| pmb0874 | C23H44NO7P | LysoPE 18:2(2n isomer)* | Lipids | LPE | - | A | 1.19E+05 | 1.02E+05 | 1.39E+05 | 4.75E+04 | 3.22E+04 | 5.13E+04 | 1.13E+00 | 3.63E-01 | -1.46E+00 | down |
| Hmpp003242 | C22H22O12 | Isorhamnetin-3-O-glucoside* | Flavonoids | Flavonols | - | A | 2.82E+06 | 2.39E+06 | 3.18E+06 | 1.20E+06 | 9.79E+05 | 9.20E+05 | 1.17E+00 | 3.70E-01 | -1.43E+00 | down |
| Lmnn002886 | C23H26O11 | 1'-O-β-D-(3,4-Dihydroxyphenethyl)-O-caffeoyl-glucoside | Phenolic acids | Phenolic acids | - | B | 4.67E+05 | 3.55E+05 | 5.68E+05 | 2.15E+05 | 1.97E+05 | 2.10E+05 | 1.13E+00 | 4.48E-01 | -1.16E+00 | down |
| Hmln002199 | C23H22O13 | Quercetin-3-O-(6''-O-acetyl)-galactoside | Flavonoids | Flavonols | - | B | 8.23E+04 | 5.38E+04 | 1.01E+05 | 3.25E+04 | 3.03E+04 | 3.68E+04 | 1.08E+00 | 4.20E-01 | -1.25E+00 | down |
| Hmxn003549 | C18H24N2O11S2 | 1,4-Dimethoxyglucobrassicin | Others | Glucosinolates | - | A | 8.81E+04 | 1.20E+05 | 1.40E+05 | 3.70E+04 | 3.04E+04 | 3.15E+04 | 1.16E+00 | 2.84E-01 | -1.82E+00 | down |
| Hmbn002692 | C22H30O14 | 6'-O-feruloylsucrose* | Phenolic acids | Phenolic acids | 118230-77-6 | A | 3.26E+05 | 3.90E+05 | 3.48E+05 | 1.51E+05 | 1.57E+05 | 1.76E+05 | 1.18E+00 | 4.55E-01 | -1.14E+00 | down |
| Lmhp010190 | C26H52NO7P | LysoPC 18:1(2n isomer) | Lipids | LPC | - | B | 1.22E+05 | 8.49E+04 | 1.29E+05 | 4.24E+04 | 3.20E+04 | 6.07E+04 | 1.07E+00 | 4.02E-01 | -1.32E+00 | down |
| Lmhn003801 | C25H24O13 | feruloylsinapoyltartaric acid* | Phenolic acids | Phenolic acids | - | B | 7.07E+04 | 5.43E+04 | 1.16E+05 | 4.01E+03 | 2.28E+03 | 1.82E+03 | 1.18E+00 | 3.37E-02 | -4.89E+00 | down |
| Cmln001958 | C19H24NO11S3- | 6-(P-hydroxybenzoic acid)-β-D-1-Glucosinolate of Methylthio--3-butenyl | Others | Glucosinolates | - | B | 3.18E+04 | 3.68E+04 | 3.46E+04 | 6.03E+03 | 7.72E+03 | 8.35E+03 | 1.18E+00 | 2.14E-01 | -2.22E+00 | down |
| pmn001672 | C23H32O15 | β-D-Furanofructosyl-α-D-(3-mustard acyl)glucoside* | Phenolic acids | Phenolic acids | - | B | 3.73E+05 | 3.77E+05 | 6.61E+05 | 1.74E+05 | 1.86E+05 | 1.58E+05 | 1.11E+00 | 3.66E-01 | -1.45E+00 | down |
| pmp000589 | C24H22O15 | Quercetin-7-O-(6'-O-malonyl)-β-D-glucoside* | Flavonoids | Flavonols | - | B | 7.90E+04 | 5.73E+04 | 9.33E+04 | 3.94E+04 | 2.87E+04 | 3.95E+04 | 1.08E+00 | 4.68E-01 | -1.09E+00 | down |
| Lmhn003802 | C26H26O14 | sinapoylsinapoyltartaric acid | Phenolic acids | Phenolic acids | - | B | 5.72E+05 | 6.86E+05 | 6.05E+05 | 1.18E+05 | 1.37E+05 | 1.31E+05 | 1.19E+00 | 2.07E-01 | -2.27E+00 | down |
| pme1540 | C28H32O16 | Isorhamnetin-3-O-neohesperidoside | Flavonoids | Flavonols | 55033-90-4 | B | 7.72E+04 | 8.58E+04 | 9.84E+04 | 3.87E+04 | 3.65E+04 | 3.73E+04 | 1.18E+00 | 4.30E-01 | -1.22E+00 | down |
| Lmjn003562 | C32H38O17 | 3,6'-O-diferuloylsucrose | Phenolic acids | Phenolic acids | 107172-40-7 | A | 4.17E+06 | 4.85E+06 | 4.63E+06 | 6.93E+05 | 9.19E+05 | 6.32E+05 | 1.19E+00 | 1.64E-01 | -2.61E+00 | down |
| Lmdp004668 | C37H38O19 | Kaempferol-O-feruloylglucosid-O-glucoside | Flavonoids | Flavonols | - | B | 1.89E+04 | 1.73E+04 | 1.11E+04 | 6.28E+03 | 6.94E+03 | 6.24E+03 | 1.12E+00 | 4.12E-01 | -1.28E+00 | down |
| pmn001536 | C36H40O20 | Tetra-O-galloyl Methyl gallate | Phenolic acids | Phenolic acids | - | B | 8.81E+04 | 1.06E+05 | 1.08E+05 | 9.00E+00 | 9.00E+00 | 9.00E+00 | 1.20E+00 | 8.92E-05 | -1.35E+01 | down |
| Lmdp004574 | C37H38O20 | Quercetin-O-feruloylglucosid-O-glucoside | Flavonoids | Flavonols | - | B | 1.80E+04 | 2.46E+04 | 2.79E+04 | 1.23E+04 | 9.63E+03 | 8.83E+03 | 1.11E+00 | 4.36E-01 | -1.20E+00 | down |
| Lmdp004696 | C38H40O20 | Kaempferol-O-sinapoylglucosid-O-glucoside | Flavonoids | Flavonols | - | A | 1.84E+04 | 2.68E+04 | 3.26E+04 | 1.10E+04 | 1.09E+04 | 1.35E+04 | 1.08E+00 | 4.56E-01 | -1.13E+00 | down |

**Supplemental Table 3 Specifically elevated metabolites in the SSD stage of CMS lines.**

| Index | Formula | Compounds | Class I | Class II | CAS | Level | A-BM1 | A-BM2 | A-BM3 | A-AM1 | A-AM2 | A-AM3 | VIP | Fold_Change | Log2FC | Type |
| --- | --- | --- | --- | --- | --- | --- | --- | --- | --- | --- | --- | --- | --- | --- | --- | --- |
| mws5037 | C9H18N2O3 | Alanylleucine | Amino acids and derivatives | Amino acids and derivatives | 1999-42-4 | A | 8.35E+05 | 8.11E+05 | 1.09E+06 | 1.88E+06 | 1.81E+06 | 1.84E+06 | 1.22E+00 | 2.02E+00 | 1.02E+00 | up |
| Lmhp001670 | C11H22N2O3 | Val-Leu | Amino acids and derivatives | Amino acids and derivatives | - | A | 3.80E+05 | 3.54E+05 | 4.93E+05 | 8.52E+05 | 8.86E+05 | 8.35E+05 | 1.22E+00 | 2.10E+00 | 1.07E+00 | up |
| Lmhp002031 | C12H24N2O3 | Leu- Leu | Amino acids and derivatives | Amino acids and derivatives | - | A | 4.61E+05 | 4.54E+05 | 5.46E+05 | 9.95E+05 | 1.10E+06 | 1.12E+06 | 1.25E+00 | 2.20E+00 | 1.13E+00 | up |
| pmb2591 | C13H14N2O3 | Acetyltryptophan | Amino acids and derivatives | Amino acids and derivatives | 2280-01-5 | A | 1.33E+05 | 1.12E+05 | 1.21E+05 | 3.41E+05 | 3.38E+05 | 5.15E+05 | 1.23E+00 | 3.26E+00 | 1.71E+00 | up |
| pme1712 | C11H20N2O6 | L-Saccharopine | Amino acids and derivatives | Amino acids and derivatives | 997-68-2 | B | 7.53E+04 | 7.97E+04 | 1.23E+05 | 2.27E+05 | 2.05E+05 | 1.85E+05 | 1.17E+00 | 2.22E+00 | 1.15E+00 | up |
| mws5035 | C15H22N2O3 | Leucylphenylalanine | Amino acids and derivatives | Amino acids and derivatives | 56217-82-4 | A | 2.09E+04 | 2.33E+04 | 2.71E+04 | 5.76E+04 | 4.58E+04 | 5.16E+04 | 1.23E+00 | 2.18E+00 | 1.12E+00 | up |
| mws0628 | C7H6O2 | 4-Hydroxybenzaldehyde | Phenolic acids | Phenolic acids | 123-08-0 | A | 3.86E+05 | 3.73E+05 | 3.69E+05 | 7.69E+05 | 7.40E+05 | 8.15E+05 | 1.26E+00 | 2.06E+00 | 1.04E+00 | up |
| mws0921 | C9H10O2 | p-Coumaryl alcohol | Phenolic acids | Phenolic acids | 3690-05-9 | A | 6.58E+04 | 6.00E+04 | 3.59E+04 | 3.31E+05 | 2.99E+05 | 3.82E+05 | 1.24E+00 | 6.26E+00 | 2.65E+00 | up |
| pme2598 | C8H8O4 | 3,4-Dihydroxybenzeneacetic acid* | Phenolic acids | Phenolic acids | 102-32-9 | A | 2.32E+05 | 2.59E+05 | 2.79E+05 | 7.50E+05 | 6.68E+05 | 7.54E+05 | 1.26E+00 | 2.82E+00 | 1.50E+00 | up |
| mws1200 | C10H10O3 | Trans-4-Hydroxycinnamic Acid Methyl Ester* | Phenolic acids | Phenolic acids | 19367-38-5 | A | 8.95E+05 | 7.38E+05 | 8.14E+05 | 2.31E+06 | 2.12E+06 | 2.00E+06 | 1.25E+00 | 2.63E+00 | 1.39E+00 | up |
| pmb2795 | C10H10O3 | 4-Methoxycinnamic acid* | Phenolic acids | Phenolic acids | 830-09-1 | A | 4.92E+05 | 4.34E+05 | 4.88E+05 | 1.72E+06 | 1.54E+06 | 1.45E+06 | 1.26E+00 | 3.34E+00 | 1.74E+00 | up |
| mad2085 | C22H18O11 | Caffeoyl-p-coumaroyltartaric acid | Phenolic acids | Phenolic acids | - | B | 9.00E+00 | 9.00E+00 | 9.00E+00 | 4.95E+04 | 4.86E+04 | 4.20E+04 | 1.27E+00 | 5.19E+03 | 1.23E+01 | up |
| Lmhn003689 | C23H20O11 | p-coumaroylferuloyltartaric acid | Phenolic acids | Phenolic acids | - | B | 1.35E+04 | 1.04E+04 | 6.42E+03 | 2.23E+04 | 1.91E+04 | 2.10E+04 | 1.10E+00 | 2.06E+00 | 1.04E+00 | up |
| Lmhn003801 | C25H24O13 | feruloylsinapoyltartaric acid* | Phenolic acids | Phenolic acids | - | B | 1.52E+04 | 1.43E+04 | 3.37E+04 | 5.88E+04 | 7.27E+04 | 7.20E+04 | 1.15E+00 | 3.22E+00 | 1.69E+00 | up |
| pmb0824 | C25H28O13 | Syringic acid O-feruloyl-O-hexoside | Phenolic acids | Phenolic acids | - | B | 1.28E+05 | 1.26E+05 | 1.25E+05 | 3.25E+05 | 3.03E+05 | 2.72E+05 | 1.26E+00 | 2.37E+00 | 1.24E+00 | up |
| pme3337 | C14H17N5O8 | N6-Succinyl Adenosine | Nucleotides and derivatives | Nucleotides and derivatives | 4542-23-8 | A | 2.65E+06 | 2.72E+06 | 3.03E+06 | 6.81E+06 | 6.67E+06 | 6.86E+06 | 1.26E+00 | 2.42E+00 | 1.28E+00 | up |
| HJN087 | C21H22O10 | Naringenin-O-glucoside* | Flavonoids | Flavonoid | - | A | 1.02E+06 | 9.63E+05 | 9.51E+05 | 4.37E+06 | 3.69E+06 | 3.34E+06 | 1.26E+00 | 3.89E+00 | 1.96E+00 | up |
| HJN090 | C21H22O10 | Butin-O-glucoside* | Flavonoids | Flavonoid | - | A | 9.42E+05 | 8.76E+05 | 9.81E+05 | 4.03E+06 | 2.92E+06 | 3.25E+06 | 1.25E+00 | 3.65E+00 | 1.87E+00 | up |
| Lmmn003398 | C23H22O12 | Kaempferol acetyl-glucoside | Flavonoids | Flavonols | - | B | 2.93E+04 | 1.60E+04 | 2.48E+04 | 1.46E+05 | 1.14E+05 | 1.30E+05 | 1.24E+00 | 5.56E+00 | 2.48E+00 | up |
| Hmln002199 | C23H22O13 | Quercetin-3-O-(6''-O-acetyl)-galactoside | Flavonoids | Flavonols | - | B | 9.07E+03 | 4.53E+03 | 6.05E+03 | 3.63E+04 | 2.17E+04 | 3.25E+04 | 1.20E+00 | 4.60E+00 | 2.20E+00 | up |
| Hmln002402 | C24H22O14 | Kaempferol-3-O-(6''-malonyl)-galactoside* | Flavonoids | Flavonols | - | B | 9.00E+00 | 9.00E+00 | 1.30E+04 | 6.40E+04 | 4.52E+04 | 6.58E+04 | 1.00E+00 | 1.35E+01 | 3.75E+00 | up |
| HJAP065 | C24H22O14 | Kaempferol-malonyl-3-O-glucoside* | Flavonoids | Flavonoid | - | A | 2.68E+04 | 1.48E+04 | 2.27E+04 | 1.42E+05 | 8.81E+04 | 1.19E+05 | 1.23E+00 | 5.43E+00 | 2.44E+00 | up |
| pmb0608 | C25H24O14 | Chrysoeriol-O-malonylhexoside | Flavonoids | Flavonoid | - | B | 9.42E+03 | 9.60E+03 | 6.17E+03 | 1.75E+04 | 2.03E+04 | 1.96E+04 | 1.19E+00 | 2.28E+00 | 1.19E+00 | up |
| pmp000589 | C24H22O15 | Quercetin-7-O-(6'-O-malonyl)-β-D-glucoside* | Flavonoids | Flavonols | - | B | 8.33E+03 | 6.91E+03 | 1.01E+04 | 2.74E+04 | 1.96E+04 | 2.08E+04 | 1.21E+00 | 2.67E+00 | 1.42E+00 | up |
| pmb0709 | C30H32O20 | Quercetin-7-O-malonylhexosyl-hexoside | Flavonoids | Flavonols | - | B | 3.43E+04 | 2.77E+04 | 2.08E+04 | 5.60E+04 | 5.51E+04 | 5.80E+04 | 1.18E+00 | 2.04E+00 | 1.03E+00 | up |
| pmf0359 | C15H16O8 | Skimmin | Lignans and Coumarins | Coumarins | 93-39-0 | B | 1.27E+05 | 1.18E+05 | 9.44E+04 | 3.78E+05 | 2.79E+05 | 2.28E+05 | 1.19E+00 | 2.60E+00 | 1.38E+00 | up |
| pma0134 | C4H8O4 | D(-)-Threose | Others | Saccharides and Alcohols | 95-43-2 | B | 4.11E+03 | 4.89E+03 | 9.32E+03 | 3.20E+04 | 3.01E+04 | 3.90E+04 | 1.21E+00 | 5.52E+00 | 2.46E+00 | up |
| pme3705 | C6H10O7 | D-Glucoronic acid* | Others | Saccharides and Alcohols | 6556-12-3 | B | 8.23E+04 | 7.45E+04 | 7.50E+04 | 2.82E+05 | 2.31E+05 | 2.23E+05 | 1.25E+00 | 3.17E+00 | 1.67E+00 | up |
| mws4174 | C8H15NO6 | N-Acetyl-β-D-mannosamine* | Others | Others | 7772-94-3 | B | 4.04E+04 | 4.02E+04 | 6.71E+04 | 9.03E+04 | 1.09E+05 | 1.16E+05 | 1.14E+00 | 2.13E+00 | 1.09E+00 | up |
| mws1038 | C11H22N2O4S | (R)-Pantetheine | Others | Others | 496-65-1 | B | 8.06E+04 | 7.50E+04 | 1.00E+05 | 2.81E+05 | 1.94E+05 | 2.87E+05 | 1.22E+00 | 2.98E+00 | 1.57E+00 | up |
| Lmfn000604 | C13H16O10 | 6-O-Galloyl-β-D-glucose* | Tannins | Tannin | 13186-19-1 | A | 8.41E+05 | 9.37E+05 | 7.54E+05 | 3.36E+06 | 2.59E+06 | 2.86E+06 | 1.25E+00 | 3.48E+00 | 1.80E+00 | up |
| HmLn000873 | C13H16O10 | 2-O-Galloyl-β-D-glucose | Tannins | Tannin | - | A | 8.22E+05 | 8.97E+05 | 8.06E+05 | 3.27E+06 | 2.85E+06 | 2.73E+06 | 1.26E+00 | 3.51E+00 | 1.81E+00 | up |
| pmb0501 | C5H14N4 | Agmatine | Alkaloids | Phenolamine | 306-60-5 | B | 4.40E+04 | 5.13E+04 | 5.49E+04 | 1.09E+05 | 8.54E+04 | 1.11E+05 | 1.21E+00 | 2.03E+00 | 1.02E+00 | up |
| mws1346 | C6H11NO4 | DL-2-Aminoadipic acid | Alkaloids | Alkaloids | 542-32-5 | B | 5.96E+05 | 5.41E+05 | 9.55E+05 | 2.12E+06 | 2.01E+06 | 1.71E+06 | 1.19E+00 | 2.79E+00 | 1.48E+00 | up |
| Lmtn002796 | C21H22O11 | Aromadendrin 7-glucoside | Terpenoids | Triterpene Saponin | - | B | 3.61E+05 | 2.83E+05 | 2.69E+05 | 1.08E+06 | 1.01E+06 | 9.04E+05 | 1.25E+00 | 3.28E+00 | 1.71E+00 | up |
| pme3009 | C6H6O6 | Trans-Citridic acid | Organic acids | Organic acids | 4023-65-8 | A | 5.07E+05 | 5.41E+05 | 6.19E+05 | 1.20E+06 | 1.09E+06 | 1.23E+06 | 1.25E+00 | 2.11E+00 | 1.08E+00 | up |
| mws1189 | C6H10O7 | D-Galacturonic acid(Gal A)* | Organic acids | Organic acids | 685-73-4 | A | 7.97E+04 | 8.94E+04 | 9.19E+04 | 2.91E+05 | 2.81E+05 | 2.72E+05 | 1.26E+00 | 3.24E+00 | 1.69E+00 | up |
| Lmmn003323 | C16H32O3 | 2-hydroxyhexadecanoic acid | Organic acids | Organic acids | 764-67-0 | B | 1.15E+06 | 1.04E+06 | 1.29E+06 | 4.45E+06 | 4.44E+06 | 4.63E+06 | 1.26E+00 | 3.88E+00 | 1.96E+00 | up |
| pmb0889 | C18H30O2 | Punicic acid | Lipids | Free fatty acids | 544-72-9 | B | 1.28E+05 | 1.53E+05 | 2.47E+05 | 4.06E+05 | 4.13E+05 | 4.15E+05 | 1.16E+00 | 2.33E+00 | 1.22E+00 | up |
| pma3606 | C18H30O3 | 9-Hydroxy-10,12,15-octadecatrienoic acid* | Lipids | Glycerol ester | - | B | 1.33E+05 | 1.56E+05 | 2.11E+05 | 4.95E+05 | 5.67E+05 | 5.88E+05 | 1.23E+00 | 3.30E+00 | 1.72E+00 | up |
| pmb2778 | C18H32O3 | 9,10-EODE* | Lipids | Free fatty acids | 65167-83-1 | B | 4.96E+05 | 5.11E+05 | 7.63E+05 | 1.42E+06 | 1.40E+06 | 1.61E+06 | 1.21E+00 | 2.50E+00 | 1.32E+00 | up |
| Rfmb091 | C18H32O3 | 9-Hydroxy-10,12-octadecadienoic acid* | Lipids | Free fatty acids | 15514-85-9 | B | 2.41E+05 | 2.52E+05 | 3.77E+05 | 7.23E+05 | 7.18E+05 | 8.41E+05 | 1.21E+00 | 2.62E+00 | 1.39E+00 | up |
| pmn001610 | C20H36O2 | Eicosadienoic acid | Lipids | Free fatty acids | 5598-38-9 | B | 3.31E+05 | 3.72E+05 | 4.74E+05 | 8.35E+05 | 8.34E+05 | 7.96E+05 | 1.22E+00 | 2.10E+00 | 1.07E+00 | up |
| pmb2804 | C18H32O4 | 13-HPODE | Lipids | Glycerol ester | 33964-75-9 | B | 1.47E+04 | 1.27E+04 | 1.50E+04 | 3.44E+04 | 3.41E+04 | 3.44E+04 | 1.26E+00 | 2.43E+00 | 1.28E+00 | up |
| Rfmb087 | C18H32O4 | 13-Oxo-9-hydroxy-10-octadecenoic acid* | Lipids | Free fatty acids | - | B | 1.92E+04 | 2.03E+04 | 3.28E+04 | 8.16E+04 | 9.39E+04 | 9.73E+04 | 1.22E+00 | 3.77E+00 | 1.92E+00 | up |

**Supplemental Table 4 Specifically down-regulated metabolites in the SSD stage of CMS lines.**

| Index | Formula | Compounds | Class I | Class II | CAS | Level | A-BM1 | A-BM2 | A-BM3 | A-AM1 | A-AM2 | A-AM3 | VIP | Fold_Change | Log2FC | Type |
| --- | --- | --- | --- | --- | --- | --- | --- | --- | --- | --- | --- | --- | --- | --- | --- | --- |
| Lmdp004426 | C38H40O21 | Quercetin-O-sinapoylglucosid-O-glucoside | Flavonoids | Flavonols | - | B | 2.99E+04 | 3.15E+04 | 2.54E+04 | 1.16E+04 | 1.14E+04 | 2.03E+04 | 1.10E+00 | 4.99E-01 | -1.00E+00 | down |
| Zmbn00584 | C12H21NO10S2 | 2-Hydroxy-4-Pentenyl Glucosinolate | Others | Glucosinolates | 19764-03-5 | A | 1.42E+07 | 1.11E+07 | 1.30E+07 | 6.65E+06 | 5.99E+06 | 6.37E+06 | 1.23E+00 | 4.95E-01 | -1.01E+00 | down |
| Lmyn002971 | C13H25NO9S2 | 3-methylpentyl glucosinolate | Others | Glucosinolates | - | A | 1.81E+06 | 1.93E+06 | 1.61E+06 | 7.96E+05 | 8.43E+05 | 9.02E+05 | 1.25E+00 | 4.75E-01 | -1.08E+00 | down |
| Cmyn102345 | C14H27NO10S3 | 6-Methylsulfinylhexyl Glucosinolate | Others | Glucosinolates | 33049-17-1 | B | 1.79E+07 | 1.79E+07 | 2.03E+07 | 8.28E+06 | 9.83E+06 | 9.46E+06 | 1.24E+00 | 4.92E-01 | -1.02E+00 | down |
| pme3007 | C9H14N2O12P2 | Uridine 5'-diphosphate | Nucleotides and derivatives | Nucleotides and derivatives | 27821-45-0 | B | 3.62E+04 | 3.56E+04 | 3.42E+04 | 1.19E+04 | 8.44E+03 | 5.58E+03 | 1.21E+00 | 2.44E-01 | -2.03E+00 | down |
| pme2117 | C10H15N5O10P2 | Adenosine 5'-Diphosphate | Nucleotides and derivatives | Nucleotides and derivatives | 58-64-0 | A | 2.39E+05 | 2.23E+05 | 2.22E+05 | 8.88E+04 | 9.98E+04 | 9.39E+04 | 1.26E+00 | 4.13E-01 | -1.28E+00 | down |
| pme1730 | C4H6O4 | D-Erythronolactone | Organic acids | Organic acids | 15667-21-7 | A | 6.13E+05 | 6.78E+05 | 7.12E+05 | 3.40E+05 | 3.31E+05 | 2.83E+05 | 1.24E+00 | 4.76E-01 | -1.07E+00 | down |
| pme2074 | C18H29NO4 | N-[(-)-Jasmonoyl]-(L)-Isoleucine (JA-L-Ile) | Organic acids | Organic acids | 120330-93-0 | A | 5.78E+05 | 4.18E+05 | 5.79E+05 | 8.65E+04 | 8.29E+04 | 7.12E+04 | 1.26E+00 | 1.53E-01 | -2.71E+00 | down |
| Xmgn006542 | C10H10O4 | Dimethyl Phthalate | Others | Others | 131-11-3 | B | 8.62E+05 | 8.20E+05 | 8.08E+05 | 2.56E+05 | 2.34E+05 | 2.30E+05 | 1.26E+00 | 2.89E-01 | -1.79E+00 | down |
| Hmln002597 | C10H9NaO4 | Sodium ferulate | Others | Others | - | A | 6.80E+05 | 6.51E+05 | 6.56E+05 | 1.93E+05 | 1.77E+05 | 1.65E+05 | 1.26E+00 | 2.69E-01 | -1.89E+00 | down |
| Lmyn002435 | C19H28O10 | Hydrangeifolin I | Others | Others | - | B | 2.99E+05 | 3.77E+05 | 3.61E+05 | 6.06E+04 | 4.86E+04 | 4.51E+04 | 1.26E+00 | 1.49E-01 | -2.75E+00 | down |
| mws0467 | C9H10O3 | 3-(4-Hydroxyphenyl)-propionic acid | Phenolic acids | Phenolic acids | 501-97-3 | B | 1.27E+05 | 1.39E+05 | 1.10E+05 | 3.64E+04 | 3.44E+04 | 5.08E+04 | 1.23E+00 | 3.23E-01 | -1.63E+00 | down |
| Lmhn102452 | C13H12O8 | Caffeoylmalic acid isomer | Phenolic acids | Phenolic acids | - | B | 8.18E+04 | 6.72E+04 | 1.15E+05 | 2.75E+04 | 4.16E+04 | 4.15E+04 | 1.14E+00 | 4.18E-01 | -1.26E+00 | down |
| mws0102 | C9H7NO2 | Indole-5-carboxylic acid* | Alkaloids | Plumerane | 1670-81-1 | B | 8.29E+05 | 7.61E+05 | 8.76E+05 | 4.43E+05 | 4.07E+05 | 3.35E+05 | 1.23E+00 | 4.80E-01 | -1.06E+00 | down |
| mws1417 | C9H7NO2 | Indole-3-carboxylic acid* | Alkaloids | Plumerane | 771-50-6 | B | 1.30E+06 | 1.23E+06 | 1.29E+06 | 6.96E+05 | 6.51E+05 | 5.47E+05 | 1.24E+00 | 4.96E-01 | -1.01E+00 | down |
| mws1090 | C6H13O9P | Glucose-1-phosphate* | Others | Saccharides and Alcohols | 59-56-3 | B | 3.13E+06 | 2.88E+06 | 3.01E+06 | 1.27E+06 | 1.42E+06 | 1.29E+06 | 1.26E+00 | 4.41E-01 | -1.18E+00 | down |
